# Impact of 17β-HSD12, the 3-ketoacyl-CoA reductase of long-chain fatty acid synthesis, on breast cancer cell proliferation and migration

**DOI:** 10.1007/s00018-019-03227-w

**Published:** 2019-07-13

**Authors:** Maria Tsachaki, Pirmin Strauss, Anja Dunkel, Hana Navrátilová, Natasa Mladenovic, Alex Odermatt

**Affiliations:** 1 Division of Molecular and Systems Toxicology, Department of Pharmaceutical Sciences, University of Basel, Klingelbergstrasse 50, 4056 Basel, Switzerland; 2 Present Address: Department of Biochemical Sciences, Faculty of Pharmacy in Hradec Králové, Charles University, Heyrovskeho 1203, 500 05 Hradec Kralove, Czech Republic

**Keywords:** Biosynthesis, Cancer, 17β-Hydroxysteroid dehydrogenase, Long-chain fatty acid, Unfolded protein response, Endoplasmic reticulum

## Abstract

Metabolic reprogramming of tumor cells involves upregulation of fatty acid (FA) synthesis to support high bioenergetic demands and membrane synthesis. This has been shown for cytosolic synthesis of FAs with up to 16 carbon atoms. Synthesis of long-chain fatty acids (LCFAs), including ω-6 and ω-3 polyunsaturated FAs, takes place at the endoplasmic reticulum. Despite increasing evidence for an important role of LCFAs in cancer, the impact of their synthesis in cancer cell growth has scarcely been studied. Here, we demonstrated that silencing of 17β-hydroxysteroid dehydrogenase type 12 (17β-HSD12), essentially catalyzing the 3-ketoacyl-CoA reduction step in LCFA production, modulates proliferation and migration of breast cancer cells in a cell line-dependent manner. Increased proliferation and migration after 17β-HSD12 knockdown were partly mediated by metabolism of arachidonic acid towards COX2 and CYP1B1-derived eicosanoids. Decreased proliferation was rescued by increased glucose concentration and was preceded by reduced ATP production through oxidative phosphorylation and spare respiratory capacity. In addition, 17β-HSD12 silencing was accompanied by alterations in unfolded protein response, including a decrease in CHOP expression and increase in eIF2α activation and the folding chaperone ERp44. Our study highlights the significance of LCFA biosynthesis for tumor cell physiology and unveils unknown aspects of breast cancer cell heterogeneity.

**Electronic supplementary material:**

The online version of this article (10.1007/s00018-019-03227-w) contains supplementary material, which is available to authorized users.

## Introduction

**Fig. 1:**
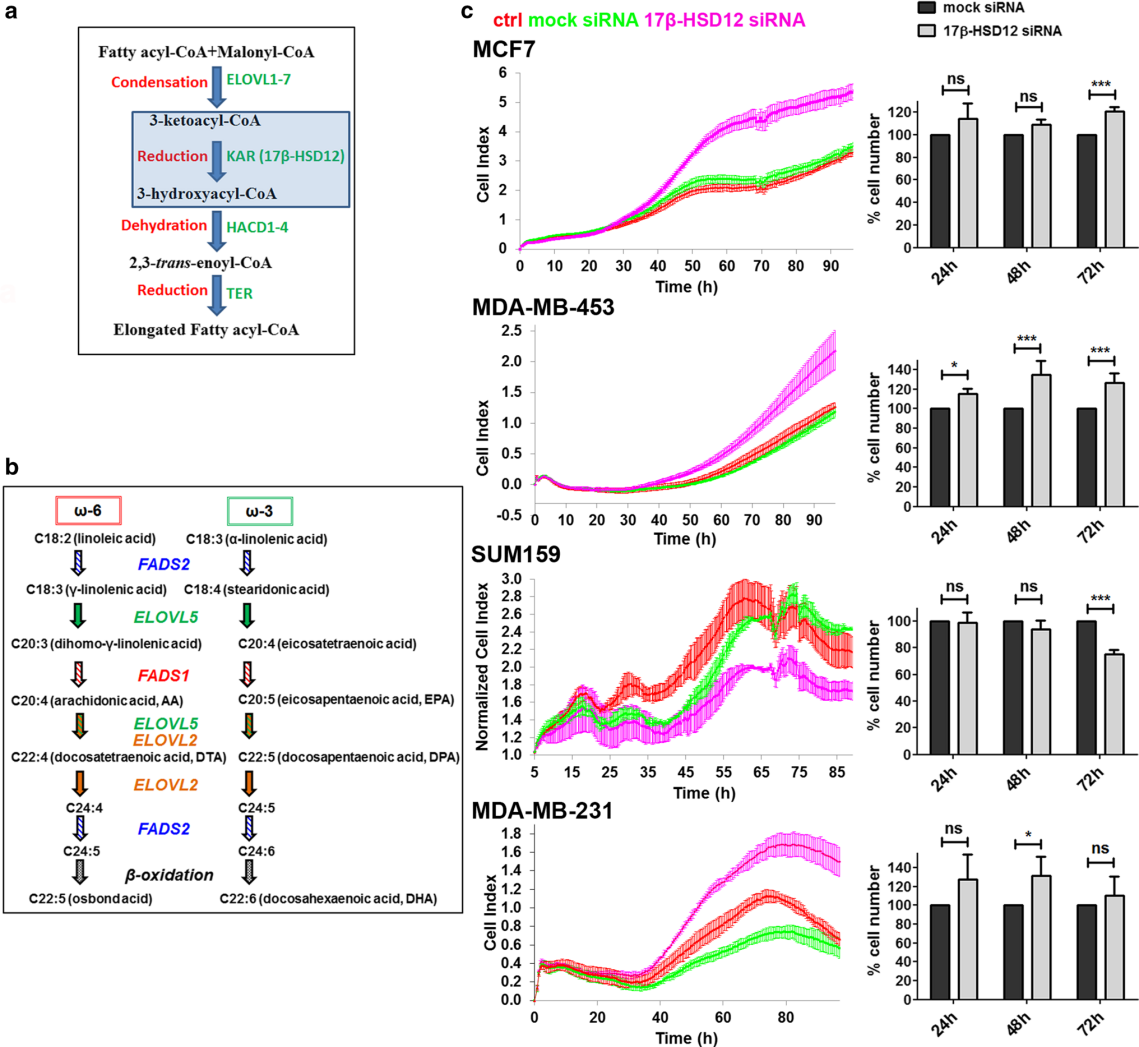
Effect of 17β-HSD12 on cancer cell proliferation. **a** Every elongation round of LCFAs consists of four steps. During the first step, the elongases of very long-chain fatty acids (ELOVL1–7) condense malonyl-CoA with a substrate fatty acyl-CoA. Subsequently, 3-ketoacyl-CoA reductase (KAR or 17β-HSD12) reduces the 3-ketoacyl-CoA to 3-hydroxyacyl-CoA, which is then subjected to dehydration by 3-hydroxyacyl-CoA dehydratases (HACD1–4). A final reduction step is performed by 2,3-*trans*-enoyl-CoA reductase (TER) to yield a fatty acyl-CoA elongated by two carbon atoms. **b** The essential PUFAs linoleic acid and α-linolenic acid give rise to a wide array of ω-6 and ω-3 FAs, respectively, through a series of elongation and/or desaturation reactions. Fatty acid desaturases (FADS1 and FADS2) and ELOVLs (ELOVL2 and ELOVL5) with distinct substrate preferences participate at different steps of this metabolic pathway. The ω-6 osbond acid (22:5) and ω-3 docosahexaenoic acid (DHA, 22:6) are produced via elongation and desaturation of docosatetraenoic acid (DTA) and docosapentaenoic acid (DPA), respectively, followed by β-oxidation in peroxisomes. **c** MCF7, MDA-MB-453, SUM159, and MDA-MB-231 cells were transfected with mock or 17β-HSD12 siRNA or left untreated (ctrl). (Left panel) Live measurements of cell proliferation were performed with the xCELLigence RTCA DP instrument. The cell index values (average and standard deviation of four technical replicates) from one out of three independent experiments are shown. For the SUM159 cells, the cell index was normalized to 5 h to account for small differences in seeding in this experiment. (Right panel) At 24 h, 48 h, and 72 h post-transfection with siRNAs, cell nuclei were stained with Hoechst-33342 and analyzed by high-content imaging. The values represent the mean and error bars SD from at least three independent experiments per cell line, and are normalized to the mock siRNA samples at each time point (*p < 0.05, ***p < 0.001, *ns* not significant)

Fatty acids (FAs) serve as energy source, act as signaling molecules, and are essential structural elements of the lipid bilayer, where they can be found in esterified form in phospholipids. The wide spectrum of FAs results from elongation of shorter precursors, through sequential addition of two carbon atoms, provided by malonyl-CoA. Production of FAs with carbon chain length up to 16 takes place in the cytosol, through a four-step process performed by the multi-activity enzyme fatty acid synthase (FASN) [1]. Long-chain fatty acids (LCFAs) containing more than 18 carbon atoms are produced in a similar fashion by four different endoplasmic reticulum (ER) enzymatic entities; the elongases of LCFAs (ELOVL1–7), 3-ketoacyl-CoA reductase (KAR) (also known as 17β-hydroxysteroid dehydrogenase type 12, 17β-HSD12 or SDR12C1), 3-hydroxyacyl-CoA dehydratases (HACD1–4) and 2,3-*trans*-enoyl-CoA reductase (TER) (Fig. 1a) [2]. FA elongation combined with desaturation leads to a multitude of distinct FAs with unique molecular properties and cellular functions.

De novo FA synthesis mainly occurs in tissues with high lipogenic activity, including the liver and adipose tissue. Nevertheless, glucose can be readily utilized by tumor cells for FA synthesis, and it was shown that the rate of lipogenesis in mouse hepatoma is comparable to that in non-neoplastic liver tissue [3]. An upregulation of de novo FA synthesis is generally accepted to have a major role in the metabolic rewiring of cancer cells [4–6]. FASN is abundantly present in human tumors, including breast cancer [7], and has been the focus of numerous anti-cancer therapeutic attempts [8]. Several other enzymes involved in FA biosynthesis and its regulation are overexpressed or overactivated in malignant tissues [9].

The role of cytosolic FA synthesis in cancer is a field of intense research; in contrast, de novo synthesis of LCFAs has received less attention. Saturated and monounsaturated LCFAs derive from elongation of palmitic acid, whereas polyunsaturated fatty acids (PUFAs) originate from two essential FAs, the linoleic acid (LA, C18:2) and α-linolenic acid (ALA, C18:3) that generate the ω-6 and ω-3 FA series, respectively [10] (Fig. 1b). ω-6 PUFAs have a double bond at position 6, when counting as 1 the carbon atom opposite of the carboxylic group, whereas ω-3 PUFAs have a double bond at the corresponding position 3. PUFAs with well-established physiological functions include the ω-6 arachidonic acid (AA) and the ω-3 docosahexaenoic acid (DHA) and eicosapentaenoic acid (EPA). Metabolism of AA gives rise to key pro-inflammatory eicosanoids, including prostaglandins and leukotrienes, which support immune system evasion and promote tumor cell proliferation and angiogenesis [11, 12]. On the other hand, DHA and EPA give rise to metabolites that dampen inflammatory response and limit malignant cell growth [13]. In many tumors, the ratio of ω-6/ω-3 PUFAs has been reported to be elevated compared to adjacent non-cancerous tissue [14–16]. Several epidemiological studies found a positive correlation between consumption of ω-3 PUFAs and reduced cancer risk [17–19]. Based on this, a wealth of in vitro, animal and clinical studies have aimed at evaluating the potential benefits of ω-3 PUFA preventive supplementation or utilization as adjuvant antineoplastic therapy [20]. Despite some promising results, so far findings have not led to definitive conclusions. This could be partially attributed to inter-individual differences in the metabolism of diet-supplemented FAs, including the expression and activity of the involved enzymes. Moreover, the possible significance of de novo biosynthesis and metabolism of LCFAs has been scarcely explored.

Interestingly, in two earlier reports, silencing of 17β-HSD12 expression, the enzyme performing the first reduction step in LCFA elongation (Fig. 1a), in one breast cancer and one ovarian cell line led to decreased proliferation, which could be reverted by AA supplementation [21, 22]. These and one additional study found a correlation between high 17β-HSD12 expression and poor prognosis for survival in patients with breast and ovarian tumors [23]. In contrast, knockdown of 17β-HSD12 in mammary epithelial cells was shown to promote cell proliferation, whereas overexpression of the enzyme led to the opposite effect [24]. Recently, an elaborate transcriptomic study of 17 different cancer types in a total of around 8000 patients was performed, with data of the Cancer Genome Atlas and Human protein Atlas Projects. This analysis showed that 17β-HSD12, along with other genes involved in the elongation and desaturation of LCFAs, correlates with either good or poor prognosis, depending on the tumor type [25]. Importantly, 17β-HSD12 was identified as a prognostic gene for favorable outcome in renal cancer and unfavorable in liver cancer patients. Thus, the role of LCFA synthesis in cancer, including that of 17β-HSD12, is insufficiently understood and deserves further research.

In the current study, we addressed the importance of this biosynthetic pathway by analyzing the consequences of 17β-HSD12 silencing in different breast cancer cell lines. This model is particularly interesting, given the heterogeneous molecular profile of breast tumor cells. We analyzed the impact of 17β-HSD12 downregulation on cell proliferation, migration, and energy metabolism. In addition, we explored whether inhibition of LCFA synthesis at the ER could constitute a stress signal for transformed cells, leading to changes in the unfolded protein response (UPR). Our results show that silencing 17β-HSD12 expression has divergent outcomes in cell survival and invasiveness in different cell lines, and leads to a number of alterations in cancer cell physiology, including energy source utilization. This study contributes to the understanding of how the biosynthetic pathway for LCFAs affects homeostasis of malignant cells, which is a necessary step for evaluating the clinical relevance of ω-3 and ω-6 PUFAs for cancer patient treatment.

## Materials and methods

### Chemicals and reagents

Trifluoromethoxy carbonyl cyanide phenylhydrazone (FCCP), AA, DHA, EPA, thioridazine (Thio), and 2,3′,4,5′-tetramethoxystilbene (TMS) were purchased from Cayman Chemical (Ann Arbor, MI, USA). All other compounds and reagents were purchased from Sigma-Aldrich (Buchs, Switzerland), unless otherwise specified.

### Cell culture and treatments

All cell lines were purchased from the American Type Culture Collection (ATCC, Manassas, VA, USA) and cultured at 37 °C and 5% CO_2_. SUM159 cells were cultured in Ham’s F12 nutrient mixture (Thermo Fisher Scientific, Waltham, MA USA), supplemented with 5% fetal bovine serum (FBS) and 5 μg/ml bovine pancreas insulin. MCF7 cells were grown in Dulbecco’s Modified Eagle’s medium (DMEM) containing 4.5 g/l glucose, 10% FBS, and nonessential amino acid mixture. For the growth of MDA-MB-453 and MDA-MB-231 cells, RPMI 1640 medium supplemented with 10% FBS was used. This medium was also used in certain experiments with SUM159 cells. For the experiments with different glucose concentrations, RPMI 1640 without glucose (Thermo Fisher Scientific, #11879020) was supplemented with 4 g/l, 2 g/l, or 0.5 g/l glucose. For the experiments to assess the possible effects of glutamine, RPMI 1640 without glutamine (Bioconcept Ltd., Allschwil, Switzerland, #1-41F01-I) was supplemented or not with 2 mM glutamine. In all culture media, 10 mM HEPES buffer pH 7.4 and a mixture of 100 U/ml penicillin/100 μg/ml streptomycin (BioConcept Ltd.) were added.

For transfection of cells with siRNAs, Lipofectamine RNAiMax (Thermo Fisher Scientific) was used. Reverse transfection was performed, with the seeding of cells and siRNA transfection taking place simultaneously. Per 100,000 cells, 20 pmol siRNA and 1 μl lipofectamine reagent was used. The target sequences recognized by the siRNAs are: mock (scrambled) 5′-UGGUUUACAUGUUUUCUGA-3′, HSD17B12 5′-GAACUAAUAUUGUCGGGAA-3′ (‘siRNA1’-used throughout the study), HSD17B12 5′-GAAAUCGGCAUCUUAGUGA-3′ (‘siRNA2’), HSD17B12 5′-UAAGAUGACACAAUUGGUA-3′ (‘siRNA3’), eIF2α 5′-GUA CAA GAG ACC UGG AUA UTT-3′ and RXRα 5′-AGG ACU GCC UGA UUG ACA ATT-3′ (Microsynth AG, Balgach, Switzerland).

In all cell treatments where compound stock solutions were dissolved in DMSO, the final concentration of the latter did not exceed 0.02%. For FA supplementation, 8 mM FAs were conjugated with 10% FA-free BSA after incubation for 1 h at 37 °C, prior to addition to the cell culture medium.

### Proliferation measurements with the real-time cell analyzer (RTCA)

Cell proliferation in real time was monitored using the xCELLigence RTCA DP instrument (ACEA Biosciences, Inc., San Diego, CA, USA). 5000 SUM159, 7500 MCF7, 10,000 MDA-MB-453, or 15,000 MDA-MB-231 cells were reverse transfected with siRNAs or left untreated and seeded on an E-plate View 16. Quadruplets of all samples were prepared and impedance signals were measured every 30 min for 96 h. Data were analyzed using the RTCA 2.0 software.

### Assessment of cell number by high-content imaging

For estimation of the cell number at different time points on 96-well plates, we fixed cells with 4% formaldehyde and stained nuclei with 5 μg/ml Hoechst-33342 (Thermo Fischer Scientific). Counting of stained nuclei was performed in high throughput with the ArrayScan^®^ high-content imaging system (Thermo Fischer Scientific), according to a protocol adjusted for each cell line. In every experiment, each sample was represented by six technical replicates (different wells) and 14 fields were randomly chosen in each well for counting.

### BrdU cell proliferation assay

To assess cell proliferation, we seeded 5000 SUM159 or 7500 MDA-MB-231 cells in 96-well plates and performed reverse transfection with siRNAs as described above. After 48 h, the conditioned medium was replaced by fresh medium-containing 10 μΜ BrdU for 2.5 h. Cells were subsequently washed twice with PBS, fixed with 4% formaldehyde, washed three times with PBS, and permeabilized with 0.2% Triton for 5 min. After three washes with PBS, DNA was denatured with 2 N HCl for 30 min, and cells were washed three times with PBS and once with PBS glycine (0.75% w/v glycine in PBS). After incubation with PBS glycine for 30 min and blocking with 1% BSA for 30 min, rat monoclonal antibody against BrdU was added (Abcam, Cambridge, UK, Ab6326) in a dilution of 1:360 in 1% BSA. Following three washes with PBS, goat anti-rat IgG Alexa Fluor 555 (Thermo Fischer Scientific) was added in a dilution of 1:360 along with 5 μg/ml Hoechst and incubated for 40 min. Cells were washed four times with PBS and cells positive for BrdU staining counted with the ArrayScan^®^ high-content imaging system (Thermo Fischer Scientific), using the BGRFR_386_23 and BGRFR_659_15 filters for detection of cell nuclei and BrdU-positive cells, respectively.

### Migration and adhesion assays

To assess migration potential, cells were transfected with siRNAs and seeded on a 35 mm dish. At 48 h post-transfection, cells were reseeded on a trans-well insert (diameter 6.5 mm, pore size 8 µm; Corning Inc., Lowell, MA, USA). The cell numbers seeded were 7500 for SUM159, 20,000 for MCF7, 30,000 for MDA-MB-453 and 15,000 for MDA-MB-231 cells. The cells were allowed to migrate for 24 h from medium-containing 1% FBS for SUM159 and MDA-MB-231 or 0.5% FBS for MCF7 and MDA-MB-453 cells towards medium-containing 10% FBS. Subsequently, cells were stained with 0.1% crystal violet (w/v in 25% methanol). After removal of the cells at the top chamber, migrated cells were visualized under the 20 × objective of a light microscope (Zeiss Axiovert 100; Carl Zeiss Microscopy GmbH, Feldbach, Switzerland). Images were captured from ten fields using a digital camera (EOS 70D, Canon, Tokyo, Japan) and cells counted with the ImageJ software (National Institute of Health, MD, USA). For the experiments where glutamine was assessed, 48 h after siRNA transfection, 40,000 MDA-MB-231 cells were seeded on a CIM-Plate 16 (ACEA Biosciences Inc.) and migration was measured over a period of 24 h using the xCELLigence RTCA DP instrument.

Cell adhesion assays were performed essentially as elaborated in Ref. [26].

### Quantitative polymerase chain reaction (qPCR)

Evaluation of mRNA abundance was performed by qPCR as described previously [26]. The sequences of the primers used are shown in Table 1.

**Table 1:** Oligonucleotide primers used for qPCR

Gene name	Sense/antisense primers (5′–3′)
*PPIA*	ATGGTCAACCCCACCGTGT/TCTGCTGTCTTTGGGACCTTGTC
*MMP1*	GCACAAATCCCTTCTACCCG/TGAACAGCCCAGTACTTATTCC
*MMP2*	ACCCATTTACACCTACACCAAG/TGTTTGCAGATCTCAGGAGTG
*MMP14*	TGCCTACCGACAAGATTGATG/ATCCCTTCCCAGACTTTGATG
*Vim*	ACCCTGCAATCTTTCAGACAG/GATTCCACTTTGCGTTCAAGG
*CTNNB1* (*β*-*cat*)	GTTCAGTTGCTTGTTCGTGC/GTTGTGAACATCCCGAGCTAG
*CHOP*	GTACCTATGTTTCACCTCCTGG/TGGAATCTGGAGAGTGAGGG
*ERp44*	AACCGGAAAGATATAGTGGCG/TCGGACAAGAGGAACACATTT
*HSD17B12*	GAACATTTCATCTGGCAGTGG/GAAGTATGGCAGGACACTCTG
*ELOVL5*	AACCTTGGACTCACACTGC/CACCAGAGGACACGGATAATC
*ELOVL7*	GCCAGCCTACCAGAAGTATTTG/CACGCAAAGACTGGAAACTG
*ELOVL2#1*	CATCTATGCACAAGTATCTTTGGTG/GGAAGATGAGACAACCGAAGG
*ELOVL2#2*	GGAGGCTACAACTTACAGTGTC/CCAGGAACTCTACTGATTTGGAG
*FADS1*	GTACAACCACCAGCACAAATAC/GAAGCGGACGTAGAAGGTAATC
*FADS2*	TGTCTACAGAAAACCCAAGTGG/TGTGGAAGATGTTAGGCTTGG
*RXRα*	CCATCGTCCTCTTTAACCCTG/AGCAAGAGCTTAGCGAACC
*CYP1B1*	CTATCACTGACATCTTCGGCG/CATACAAGGCAGACGGTCC
*CYP4V2*	GGCTTGATCTCTGGTACCTTATG/GCCATCACCTCTACAGTCTTC
*PTGER2*	CCTCATTCTCCTGGCTATCATG/CTTTCGGGAAGAGGTTTCATTC
*GADD34*	GAAACCCCTACTCATGATCCG/AAATGGACAGTGACCTTCTCG

### Western blotting

Cell lysis and sample preparation for western blot have been described before [27]. The antibodies used for detection of specific proteins and their final concentrations in 5% defatted milk blocking solution were the following.

17β-HSD12 0.05 μg/ml (SIGMA-Aldrich HPA016427), α-tubulin 0.062 μg/ml (GeneTex, Irvine, CA, USA, #GTX628802), actin 0.67 μg/ml (Santa Cruz Biotechnology, sc-1616), PPIA 0.05 μg/ml (Abcam, Ab41684), PERK 0.1 μg/ml (Cell signaling Technology, Danvers, MA, USA, #3192), eIF2α 0.1 μg/ml (Cell Signaling Technology, #9722S), peIF2α 0.3 μg/ml (Cell Signaling Technology, #119A11), ATF4 0.1 μg/ml (Cell Signaling Technology, #11815S), ATF6 0.25 μg/ml (Cell Signaling Technology, #65880S), CHOP 1.2 μg/ml (Cell Signaling Technology, #2895S), sXBP1 0.1 μg/ml (Cell Signaling Technology, #12782S), GRP78 0.62 μg/ml (BD Bioscience, San Jose, CA, USA, #610978), ERp44 1:1000 dilution [26], PDI 0.1 μg/ml (Abcam, Cambridge, UK, Ab2792), ERp72 1:1000 dilution (Stressgen), GRP94 1:1000 dilution [28], FADS1 0.5 μg/ml (Santa Cruz Biotechnology, sc-134337), FADS2 0.5 μg/ml (Abcam, ab72189), G6PD 1 μg/ml (Bethyl Laboratories, TX, USA, A300-404A), PGD 1:1000 (Abcam, ab129199) and RXRα 0.1 μg/ml (Santa Cruz Biotechnology, sc-515929), Akt 0.1 μg/ml (Abcam, ab126811), pAkt Ser473 0.1 μg/ml (Cell Signaling Technology, #9271), Caspase-9 (Cas-9)/cleaved Cas-9 0.1 μg/ml (Cell Signaling Technology, #9508), Caspase-3 (Cas-3) 0.1 μg/ml (Cell Signaling Technology, #9665), cleaved Cas-3 0.1 μg/ml (Cell Signaling Technology, #9664), Bcl-2 0.1 μg/ml (Cell Signaling Technology, #2870), Bax 0.1 μg/ml (Cell Signaling Technology, #5023), and ELOVL5 1 μg/ml (Origene, #TA315700). Samples were not boiled for detection of ELOVL5, due to the protein aggregation occurring otherwise. The signal density of protein bands was measured using the ROI manager tool of ImageJ. For relative protein-level quantification, the density of each band was compared to that of the loading control (actin, α-tubulin or PPIA) in every lane. Different loading controls were used depending on the size of the proteins of interest and the species origin of the primary antibody.

### Immunofluorescence staining and microscopy

Indirect immunofluorescence staining has been described earlier [26]. Visualization of the fluorescence signal and image acquisition was performed utilizing the 20 × objective of a DMI4000 B Microscope (Leica Microsystems, Wetzlar, Germany) and processed with the ImageJ software.

### Seahorse XF Cell Mito Stress Test

Measurements of oxygen consumption rate (OCR) and extracellular acidification rate (ECAR) were performed in real time with the Seahorse XF96 analyzer (Agilent Technologies, CA, USA). The different parameters of mitochondrial respiration were evaluated using the Seahorse XF Cell Mito Stress Test, according to the manufacturer’s instructions. Cells were transfected 48 h prior to the assays with siRNAs and seeded in six replicates per sample on a Seahorse XF96 V3 PS Cell Culture Microplate (Agilent Technologies). In this assay, baseline cellular OCR was measured, representing both mitochondrial and non-mitochondrial respiration (Suppl. Figure 7a). The proportion of mitochondrial OCR linked with ATP production is measured after addition of the ATPase inhibitor oligomycin at a concentration of 1 μΜ. Subsequently, 0.5 μM of the uncoupling agent FCCP is added, which collapses the electron gradient across the mitochondrial membrane and leads to a compensatory maximal oxygen consumption from the mitochondria, thereby enabling measurement of maximal respiration. The spare respiratory capacity is calculated by deduction of the basal mitochondrial respiration from this value. Finally, a mixture of 0.5 μM of antimycin and 0.5 μM rotenone is added, inhibiting function of complex III and I, and thus, completely shutting down mitochondrial oxygen consumption. This allows for calculation of non-mitochondrial OCR. Deduction of this value from that obtained after oligomycin treatment yields the portion of OCR resulting from proton leak. Basal respiration is the initial OCR value excluding non-mitochondrial respiration. Eto or Thio was added at a concentration of 40 μΜ 15 min prior to the commencement of the assay. Following the measurements, values were normalized to the protein content of each sample using the Pierce™ BCA Protein Assay Kit (Thermo Fisher Scientific, MA, USA). Data were analyzed employing the Wave 2.6.0 software.

### Quantification of AA

To quantify AA in cells, the competitive AA ELISA assay was used (OKEH02583, Aviva Systems Biology, CA, USA), in line with the manufacturer’s guidelines. AA was measured in transfected cells 48 h post-siRNA delivery. Cells were subjected to five freeze/thaw cycles to break up cell membranes, and samples were centrifuged for 20 min at 1200*g*, 4 °C. Subsequently, the supernatant was collected and assayed on the ELISA plate. The amount of AA was calculated based on the standard curve of serially diluted standards provided with the kit. To normalize to the protein content of each sample, the Pierce™ BCA Protein Assay was employed.

### Quantitative and statistical analysis

All experiments in this study were performed at least three times independently and values shown in graphs are the mean of these experiments (with the exception of xCELLigence experiments where a representative measurement is shown in Fig. 1c). Error bars represent standard deviations (SD). Statistical significance of the difference between conditions was calculated with a two-tailed Student’s *t* test. The levels of significance are as follows: *p < 0.05, **p < 0.01, and ***p < 0.001.

## Results

### Effect of 17β-HSD12 downregulation on breast cancer cell proliferation and migration

To assess the impact of 17β-HSD12 on proliferation and migratory potential of tumor cells, we used four breast cancer cell lines, each representing one of the three main molecular subtypes of breast cancer; the MCF7 that is positive for the estrogen receptor-α (ER) and progesterone receptor (PR) but negative for the human epidermal growth factor receptor 2 (HER2), the MDA-MB-453 that is negative for ER and PR but positive for HER2, and the SUM159 and MDA-MB-231 that are triple-negative (ER^−^, PR^−^, HER2^−^). First, we evaluated the efficiency of siRNA knockdown over time (24 h, 48 h, and 72 h) in all cell lines by western blot, using a specific antibody against 17β-HSD12. In all cases, we found a time-dependent decrease in protein levels after siRNA transfection compared to mock- and non-transfected cells (Suppl. Figure 1). Next, we tested the effect of 17β-HSD12 downregulation on cell proliferation with live measurements up to 96 h after siRNA transfection using the xCELLigence RTCA (Fig. 1c, left panel). In parallel, we performed endpoint measurements of cell numbers at 24 h, 48 h, and 72 h, by Hoechst-33342 staining of nuclei and high-content imaging analysis (Fig. 1c, right panel). The results from both approaches were in accordance. Downregulation of 17β-HSD12 led to decreased proliferation of SUM159 cells and increased proliferation of MCF7, MDA-MB-453 and MDA-MB-231 cells. The time after knockdown when differences in proliferation were statistically significant varied, which can partly be attributed to the different growth rates among cell lines. To validate the specificity of the siRNA against 17β-HSD12 used in the above experiments (‘siRNA1’), we employed two additional siRNAs (‘siRNA2’ and ‘siRNA3’) in experiments with Hoechst-33342 staining and high-content imaging for the SUM159 and MDA-MB-231 cells and obtained similar results (Suppl. Figure 2).

Subsequently, we explored whether the reduction in cell number following 17β-HSD12 knockdown in SUM159 cells could be due to increased apoptosis. We found unchanged ratios of cleaved Cas-9/Cas-9, as well as cleaved Cas-3/Cas-3 at 48 h post-transfection with 17β-HSD12 siRNA (Suppl. Figure 3a). In addition, the ratio of the apoptosis regulators Bax/Bcl-2, which is used as an indicator of apoptotic levels, was not altered (Suppl. Figure 3a). Moreover, we examined whether ferroptosis, a non-apoptotic form of cell death involving iron-dependent accumulation of lipid reactive oxygen species, could contribute to the reduced cell number after 17β-HSD12 downregulation in SUM159 cells. For this purpose, we treated cells with 1 μΜ of the ferroptosis inhibitor ferrostatin and knocked-down 17β-HSD12. Our data showed a significant reduction in cell number upon ferrostatin treatment, as observed in the absence of the inhibitor (Suppl. Figure 3b). Taken together, the above results do not support an involvement of apoptosis or ferroptosis as mechanisms implicated in the 17β-HSD12-knockdown phenotype in SUM159 cells.

**Fig. 2:**
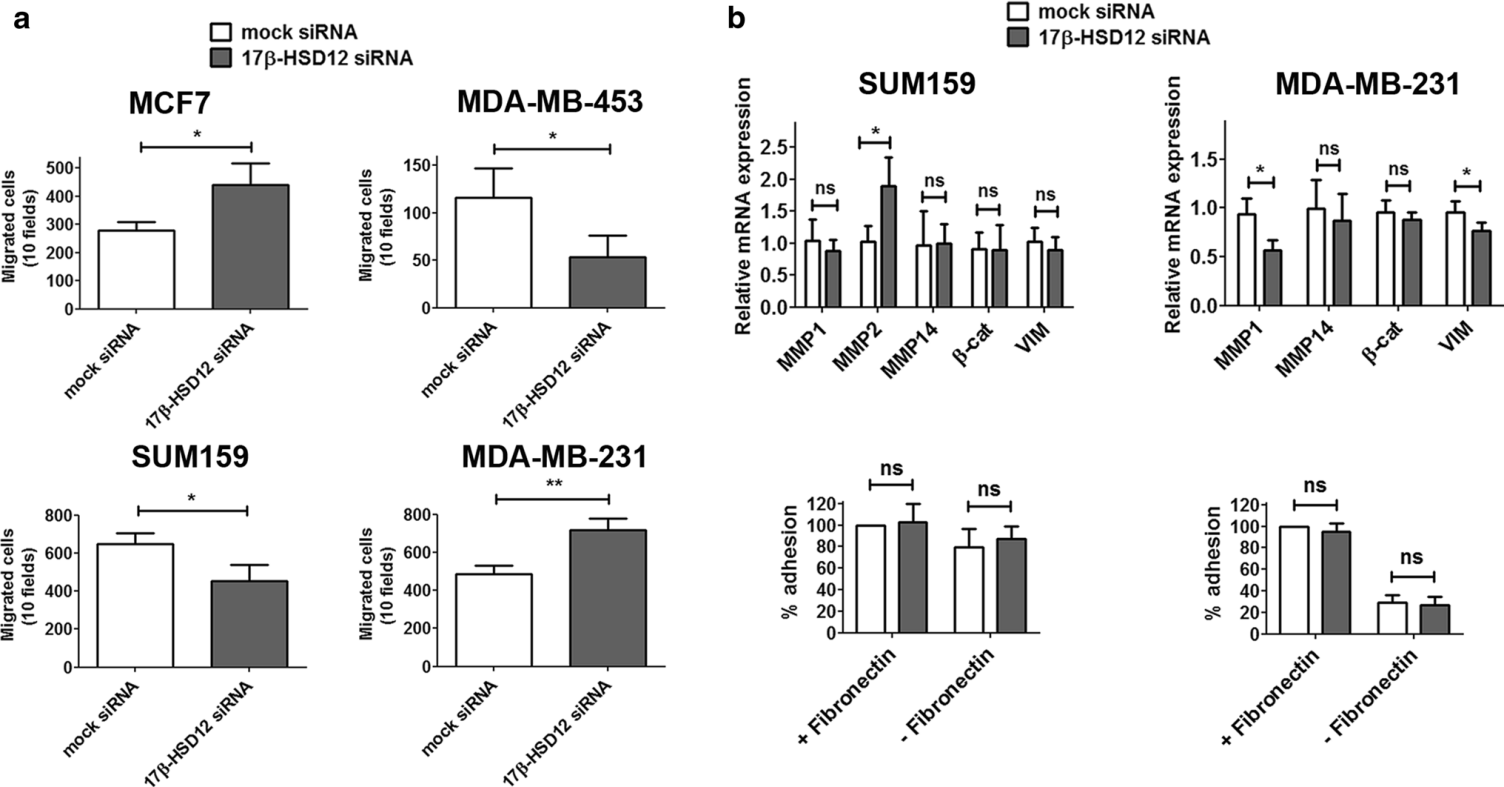
Impact of 17β-HSD12 silencing on cancer cell migration and adhesion. **a** Trans-well migration assays for MCF7, MDA-MB-453, SUM159, and MDA-MB-231 cells after transfection with mock or 17β-HSD12 siRNAs. The absolute values of migrated cells counted are shown (mean ± SD, *n* = 3, *p < 0.05, **p < 0.01). **b** (top) Relative mRNA levels of the indicated genes measured with qPCR 48 h after 17β-HSD12 downregulation in SUM159 or MDA-MB-231 cells (mean ± SD, *n* = 4, *p < 0.05, *ns* not significant) (bottom). The ability of SUM159 or MDA-MB-231 cells to adhere to plates, with or without fibronectin coating, was tested in mock- or 17β-HSD12-siRNA-transfected cells (mean ± SD, *n* = 6, *ns* not significant)

We then examined the impact of 17β-HSD12 knockdown on cell migration using the trans-well migration assay with serum as an attractant to promote cell mobility (Fig. 2a). We observed that MCF7 and MDA-MB-231 cells exhibited increased migratory capacity upon 17β-HSD12 silencing, whereas SUM159 cells displayed a decrease. These results follow the behavior of the proliferation phenotype of 17β-HSD12-knockdown cells. However, MDA-MB-453 cells showed higher proliferation after 17β-HSD12 downregulation, but decreased migration. Since this cell line is hardly invasive (four times more cells than those seeded for the SUM159 cells and lower FBS concentration in the top chamber were used in the assay), the physiological relevance of this result is unclear. Given that the cell cytoskeleton constitutes a major component of cell motility, we then investigated whether the observed alterations in cell migration are reflected in the subcellular architecture of two vital cytoskeletal elements; actin filaments and microtubules. For these experiments, we compared SUM159 and MDA-MB-231 cells and observed a decrease and increase, respectively, in cell growth and migration following 17β-HSD12 knockdown. Immunofluorescence staining with phalloidin-FITC showed that the actin cytoskeleton outlines cells with less elongated and smoother morphology after 17β-HSD12 downregulation in MDA-MB-231 cells, whereas no difference was observed in SUM159 cells (Suppl. Figure 4). However, staining of microtubules with an antibody against α-tubulin in SUM159 cells showed that 17β-HSD12 silencing caused redistribution of the microtubular network around the nucleus (Suppl. Figure 5). No obvious difference in α-tubulin pattern was observed between mock- and 17β-HSD12-siRNA-transfected MDA-MB-231 cells.

We further investigated the changes accompanying the altered migration in SUM159 and MDA-MB-231 cells after 17β-HSD12 knockdown. Specifically, we analyzed the mRNA expression levels of genes involved in cell migration and adhesion during cancer metastasis. In SUM159 cells, an increase in matrix metalloproteinase 2 (MMP2) was observed after 17β-HSD12 silencing (Fig. 2b). Although no expression of MMP2 could be detected in MDA-MB-231 cells, 17β-HSD12 knockdown led to decreased expression of MMP1 and of the intermediate filament protein vimentin (VIM). One would expect that these changes lead to decreased adhesion in SUM159 cells due to increased MMP2 levels and increased adhesion in MDA-MB-231 cells due to decreased MMP1 and VIM levels. However, in cell adhesion assays using fibronectin-coated dishes, no difference in cell adhesion was observed after 17β-HSD12 downregulation in neither cell line (Fig. 2b).

### Investigation of the differential impact of 17β-HSD12 on proliferation and migration of SUM159 and MDA-MB-231 cells

**Fig. 3:**
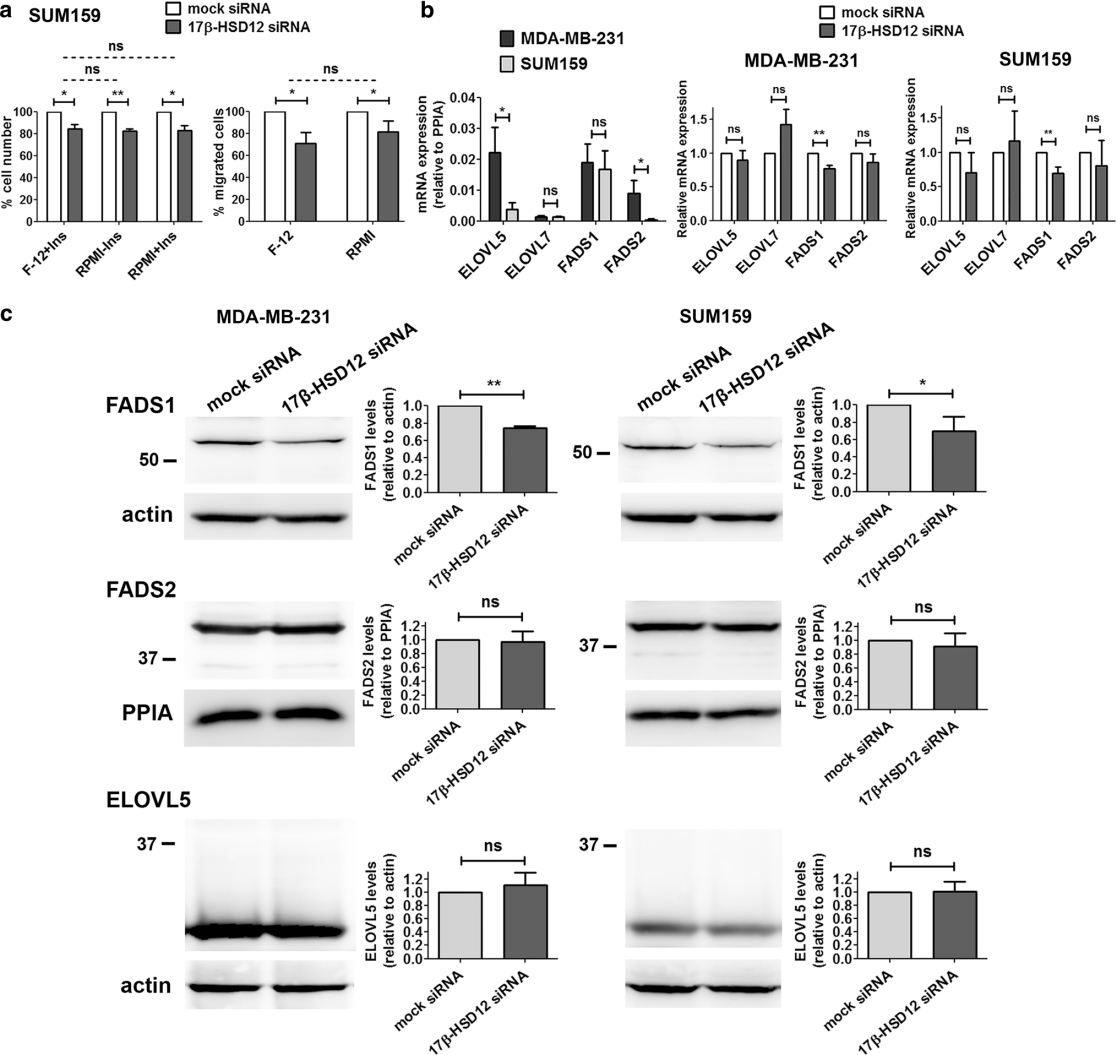
Possible determinants of the differential changes after 17β-HSD12 downregulation in SUM159 and MDA-MB-231 cells. **a** (Left panel) SUM159 cells were cultured in Ham’s F12 nutrient mixture with insulin, or RPMI 1640 with or without insulin. 17β-HSD12 expression was downregulated with siRNAs and cell number was determined 72 h later. (Right panel) Trans-well migration assay after 17β-HSD12 silencing in SUM159 cells grown in Ham’s F12 nutrient mixture with insulin or RPMI 1640 without insulin. The statistical analyses compare mock- and 17β-HSD12-siRNA samples in each case (solid lines), as well as the difference between the mock-17β-HSD12-siRNA groups under different culture conditions (dotted lines) (mean ± SD, *n* = 3, *p < 0.05, **p < 0.01, *ns* not significant). **b** (left panel) mRNA expression relative to PPIA of the indicated genes in MDA-MB-231 and SUM159 cells. (Middle and right panels) mRNA expression of elongase (ELOVL5 and ELOVL7) and desaturase (FADS1 and FADS2) genes 48 h after 17β-HSD12 downregulation in MDA-MB-231 and SUM159 cells (mean ± SD, *n* = 4, *p < 0.05, **p < 0.01, *ns* not significant). **c** The expression of FADS1, FADS2 and ELOVL5 was analyzed by western blot 48 h after 17β-HSD12 downregulation in MDA-MB-231 and SUM159 cells. A representative blot (left panel) and analysis of band density from at least three independent experiments (right panel) is shown. Values are normalized to mock siRNA samples (mean ± SD, *p < 0.05, **p < 0.01, *ns* not significant)

We next addressed the underlying reason for the diverse phenotypes observed after 17β-HSD12 downregulation. To compare closely related cell systems, we used SUM159 and MDA-MB-231 cells. Interestingly, despite both being triple-negative breast cancer cell lines, SUM159 and MDA-MB-231 cells exhibited decreased and increased cell proliferation and migration, respectively, after 17β-HSD12 knockdown. Since 17β-HSD12 is involved in LCFA synthesis, we reasoned that the difference could lie in the availability of precursors in the culture medium. Of note, the Ham’s F-12 nutrient mixture (hereinafter F-12), used to culture SUM159 cells, contains the AA precursor linoleic acid, which is not present in the RPMI 1640 medium for MDA-MB-231 cells. This could mean that in SUM159 cells, 17β-HSD12 actively produces AA, which, as mentioned above, has been shown to promote cell growth and invasion. In this case, knockdown of 17β-HSD12 would lead to the observed reduction of cell growth and migratory potential, because of reduced AA production. In contrast to our hypothesis, culturing SUM159 cells in the RPMI 1640 medium neither altered the effect of 17β-HSD12 downregulation on cell number nor migration (Fig. 3a), suggesting that the culture medium is not responsible for the distinct phenotypes after 17β-HSD12 silencing in SUM159 and MDA-MB-231 cells. For culturing SUM159 cells, the F-12 medium is also supplemented with insulin [29]. However, supplementation of RPMI 1640 with insulin did not change the 17β-HSD12-knockdown effect on cell proliferation.

After excluding the culture medium as a determining factor, we compared the expression in the two cell lines of the different ELOVL enzymes (ELOVL2, ELOVL5, and ELOVL7) and the fatty acid desaturases (FADS1 and FADS2), since these enzymes preferentially catalyze distinct steps during PUFA biosynthesis (Fig. 1b). We observed that the mRNA and protein expression of ELOVL5 was significantly lower in the SUM159 cells (Fig. 3b, c). Although FADS2 mRNA levels were higher in MDA-MB-231 compared to SUM159 cells, protein expression was comparable in both cell lines (Fig. 3b, c). The mRNA of ELOVL7 that is also able to elongate certain LCFAs in a tissue-restricted manner is present at equally low levels in both cell lines. Interestingly, we could not detect any mRNA or protein expression of ELOVL2 in neither cell line. Downregulation of 17β-HSD12 did not lead to changes in expression of the analyzed enzymes, with the exception of decreased FADS1 mRNA and protein levels in both cell lines (Fig. 3b, c), which may further accentuate the 17β-HSD12-knockdown phenotype.

### Rescue of the effect of 17β-HSD12 silencing on cancer cell proliferation and migration

**Fig. 4:**
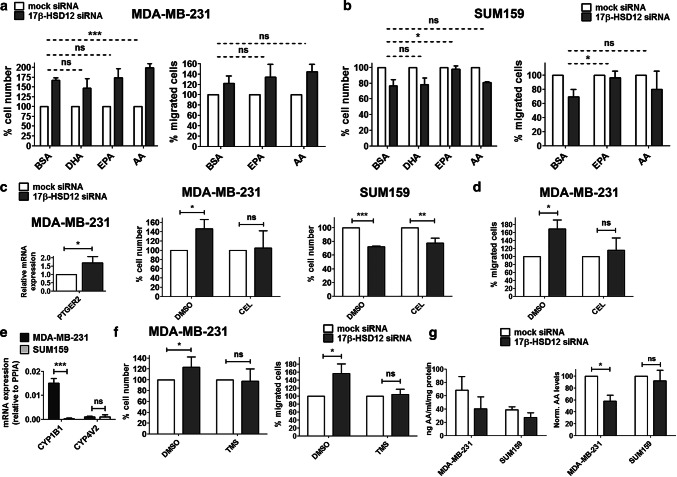
Fatty acids involved in the 17β-HSD12-knockdown phenotype. In MDA-MB-231 (**a**) and SUM159 (**b**) cells, cell number was determined after 17β-HSD12 knockdown and supplementation with BSA or BSA-conjugated DHA, EPA, and AA. The number of cells that migrated in a trans-well assay was counted after silencing of 17β-HSD12 expression and treatment with BSA or BSA-conjugated EPA and AA. The statistical analysis shown compared the degree of difference between the various mock and 17β-HSD12 siRNA groups (mean ± SD, *n* = 4, *p < 0.05, **p < 0.01, *ns* not significant). **c** PTGER2 mRNA expression was measured in MDA-MB-231 cells 48 h after 17β-HSD12 downregulation (mean ± SD, *n* = 4, *p < 0.05). No expression of PTGER2 could be detected in SUM159 cells. The number of MDA-MB-231 and SUM159 cells was assessed in mock- or 17β-HSD12-siRNA-transfected cells after treatment with DMSO or 1 μΜ of the Cox-2 inhibitor celecoxib (CEL) (mean ± SD, *n* = 3, *p < 0.05, **p < 0.01, ***p < 0.001, *ns* not significant). **d** The number of migrated cells was evaluated for MDA-MB-231 cells following 17β-HSD12 silencing and treatment with DMSO or CEL (mean ± SD, *n* = 3, *p < 0.05, *ns* not significant). **e** mRNA levels of the indicated genes in MDA-MB-231 and SUM159 cells (mean ± SD, *n* = 4, ***p < 0.001, *ns* not significant). **f** Cell number and migrating cells for MDA-MB-231 cells following treatment with DMSO or the CYP1B1 inhibitor TMS and mock or 17β-HSD12 siRNA transfection (mean ± SD, *n* = 3, *p < 0.05, *ns* not significant). **g** (Left panel) amount of AA (ng/ml/mg of protein) in extracts from mock- or 17β-HSD12-siRNA-transfected MDA-MB-231 (*n* = 3) and SUM159 cells (*n* = 4), as measured with a competitive ELISA assay. (Right panel) AA levels normalized to mock siRNA samples (mean ± SD, *p < 0.05, *ns* not significant)

Next, to test whether a deficiency in production of different LCFAs upon 17β-HSD12 silencing could account for the differential knockdown phenotypes, we downregulated the enzyme in the two cell lines and supplemented the medium with BSA-conjugated AA, EPA, and DHA, the FAs with the most well-studied role in cancer cell growth. We found that supplementation of AA in MDA-MB-231 cells led to a significantly greater increase in cell number and number of proliferating BrdU-positive cells at 48 h after 17β-HSD12-knockdown (Fig. 4a; Suppl. Figure 6a and 6b). Although a similar trend of AA was observed on the migration ability of MDA-MB-231 cells after 17β-HSD12 downregulation, the difference compared to BSA-treated cells did not reach statistical significance. EPA and DHA supplementation did not affect the 17β-HSD12-knockdown proliferation phenotype, and EPA supplementation did not alter cell migration. In contrast, a complete rescue of decreased cell number 72 h after 17β-HSD12 knockdown, and a decreased number of actively proliferating cells 48 h following 17β-HSD12 downregulation, was observed upon EPA supplementation in SUM159 cells (Fig. 4b; Suppl. Figure 6a and 6b). Although AA treatment slightly reverted the number of proliferating cells 48 h after 17β-HSD12 knockdown (Suppl. Figure 6b), it failed to rescue the reduced cell number observed 72 h following 17β-HSD12 silencing (Fig. 4b; Suppl. Figure 6a). Importantly, EPA was equally able to rescue the reduction in cell migration following 17β-HSD12 silencing in SUM159 cells (Fig. 4b). The above results imply that in MDA-MB-231 cells, AA under normal conditions sustains cancer cell growth, possibly through downstream metabolism towards known mediators of inflammation that promote cell survival.

One of the most extensively investigated AA metabolites is prostaglandin E2 (PGE2), which is produced through the enzymatic function of cyclooxygenase 2 (Cox2). It is possible that in MDA-MB-231 cells, 17β-HSD12 antagonizes this pathway of AA metabolism by further elongating it towards longer FAs that do not support growth and migration. Interestingly, we found that the PGE2 cell surface receptor 2 (PTGER2) is expressed in MDA-MB-231 but not SUM159 cells and its levels are significantly upregulated after 17β-HSD12 downregulation (Fig. 4c). We could not detect mRNA expression of other PGE2 receptors (PTGER1, PTGER3, and PTGER4) in MDA-MB-231 or SUM159 cells. Subsequently, we examined whether the Cox2 pathway of AA metabolism could be responsible for the increased proliferation upon 17β-HSD12 silencing in MDA-MB-231 cells by treating mock- or 17β-HSD12 siRNA-transfected cells with the selective inhibitor Celecoxib (CEL). CEL treatment reverted the increased proliferation due to 17β-HSD12 downregulation in MDA-MB-231 cells (Fig. 4c; Suppl. Figure 6c). In contrast, CEL treatment had no effect on the decreased proliferation of SUM159 cells after 17β-HSD12 knockdown, supporting that this pathway does not mediate the effect of 17β-HSD12 downregulation in this cell line (Fig. 4c; Suppl. Figure 6c). Furthermore, CEL was able to inhibit the increased migration triggered by 17β-HSD12 knockdown in the MDA-MB-231 cells (Fig. 4d).

We then explored whether other pathways of AA metabolism are involved in the increased proliferation after 17β-HSD12 silencing in MDA-MB-231 cells by analyzing mRNA expression of the responsible enzymes. First, we tested the lipoxygenase enzymes (LOX5, LOX12, and LOX15) through which leukotrienes are produced. We did not find mRNA expression of any LOX enzyme in MDA-MB-231 or SUM159 cells, with the exception of LOX5 that was expressed at low levels in both. We then tested for expression of CYP450 enzymes, which convert AA to a plethora of biologically active metabolites, including epoxyeicosatetraenoic acids (EETs) and hydroxyeicosatetraenoic acids (HETEs). Among the CYP450 enzymes analyzed (CYP1A2, CYP2J2, CYP4V2, CYP1B1, and CYP4F22), we detected measurable mRNA levels of CYP4V2 and CYP1B1. CYP4V2 was expressed at comparable levels in the two cell lines, but CYP1B1 mRNA was substantially expressed only in MDA-MB-231 cells, suggesting a possible role of this enzyme in the observed effects (Fig. 4e). To test whether CYP1B1 could mediate the increase in proliferation and migration induced after 17β-HSD12 downregulation in MDA-MB-231 cells, we used the CYP1B1 inhibitor TMS. TMS treatment abolished the 17β-HSD12 downregulation-dependent increase in proliferation and migration of MDA-MB-231 cells (Fig. 4f). AA incorporated into membrane phospholipids has been shown to promote proliferation through the Akt survival pathway. Unexpectedly, knockdown of 17β-HSD12 in MDA-MB-231 cells decreased the ratio of activated Akt phosphorylated on Ser473 to total Akt levels (Suppl. Figure 6d). Intriguingly, measurement of AA levels with a competitive ELISA assay showed that the levels of cellular AA are lower in SUM159 compared to MDA-MB-231 cells (Fig. 4g). Furthermore, 17β-HSD12 knockdown did not alter AA levels in SUM159, while it decreased its levels in MDA-MB-231 cells. This could be due to upregulation of AA processing by COX and CYP enzymes to downstream metabolites, which supports the results of the previously described findings (Fig. 4c, d, f).

### Impact of 17β-HSD12 silencing on energy metabolism

**Fig. 5:**
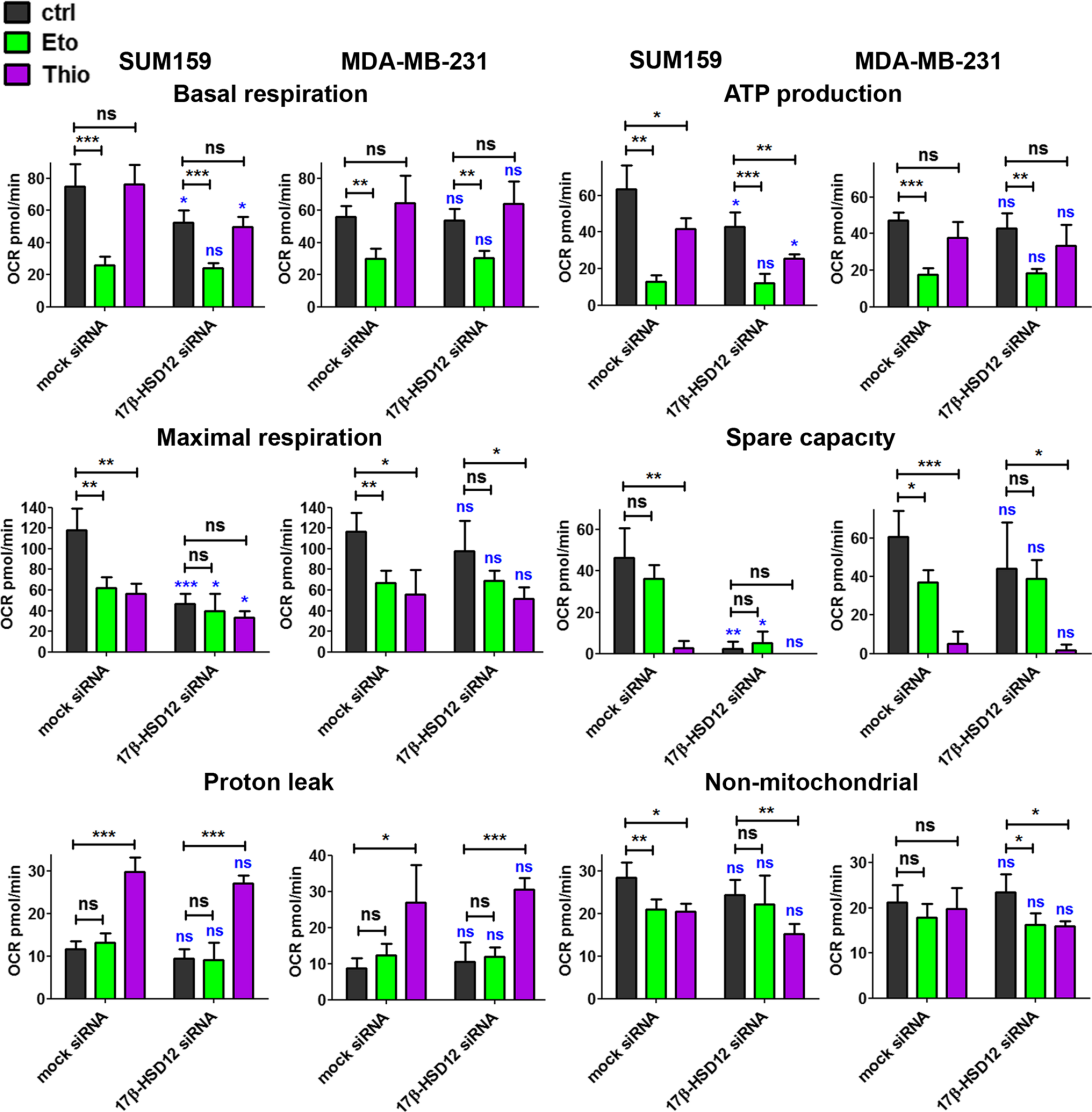
Influence of 17β-HSD12 silencing on oxygen consumption. OCR was measured in real time using the Seahorse XF96 analyzer, 48 h after 17β-HSD12 downregulation in SUM159 and MDA-MB-231 cells. Different parameters of mitochondrial function (basal respiration, ATP production, maximal respiration, spare respiratory capacity, and proton leak), as well as non-mitochondrial oxygen consumption were calculated employing the Seahorse XF Cell Mito Stress Test. Samples were left untreated (ctrl) or treated with the β-oxidation inhibitors Eto (mitochondrial) or Thio (peroxisomal). The statistical analysis depicted in blue characters compared the differences in the values between the corresponding mock siRNA and 17β-HSD12 siRNA samples. The rest of the statistical analysis shown in black letters was performed to study the differences between the indicated groups (mean ± SD, *n* = 5, *p < 0.05, **p < 0.01, ***p < 0.001, *ns* not significant)

We subsequently examined whether inhibition of the biosynthetic pathway for LCFAs can trigger metabolic rewiring in cancer cells. This could be a direct consequence of utilization of these FAs as energy sources or indirect through the pleiotropic effects of LCFAs on cancer cell physiology. We first measured in real-time OCR, indicative of one of the main pathways for energy production in cells, mitochondrial oxidative phosphorylation (OXPHOS). For comparison and potential correlation with the proliferation phenotypes observed after 17β-HSD12 silencing, we used in these experiments MDA-MB-231 and SUM159 cells both grown in RPMI 1640 medium. We first measured mitochondrial respiration using the Seahorse Mitostress test (see materials and methods), where a sequential addition of stressors (oligomycin, FCCP, and antimycin/rotenone) allows calculating the different parameters of mitochondrial respiration (basal respiration, ATP production, maximal respiration, spare capacity, and proton leak), as well as the part of OCR attributed to non-mitochondrial oxygen consumption. LCFAs are first oxidized in peroxisomes, and after reaching a certain length can be transferred to mitochondria for further breakdown [30]. To assess the potential contribution of FA oxidation in the measured OCR, we used Etomoxir (Eto), an irreversible inhibitor of carnitine palmitoyltransferase 1 that blocks mitochondrial β-oxidation, and the specific inhibitor of peroxisomal β-oxidation Thio [31, 32]. The experiments were performed 48 h post-transfection, a time point when the difference in proliferation between mock- and 17β-HSD12-siRNA-transfected cells is not yet evident in SUM159 cells. Our results showed that the baseline OCR, ATP production, maximal respiration, and spare respiratory capacity were significantly reduced after 17β-HSD12 silencing in SUM159 but not in MDA-MB-231 cells (Fig. 5; Suppl. Figure 7a). Proton leak and non-mitochondrial respiration were not affected in neither cell line. Eto treatment significantly reduced basal respiration, mitochondrial ATP production, and maximal respiration in both cell lines, demonstrating that β-oxidation is an overriding factor for these cancer cells. Treatment with Thio reduced ATP production in SUM159 but not MDA-MB-231 cells. However, a dramatic decline in maximal respiration and spare respiratory capacity is observed in both cell lines after Thio treatment. Interestingly, Thio but not Eto treatment leads to a significant rise in proton leak in both cell lines.

**Fig. 6:**
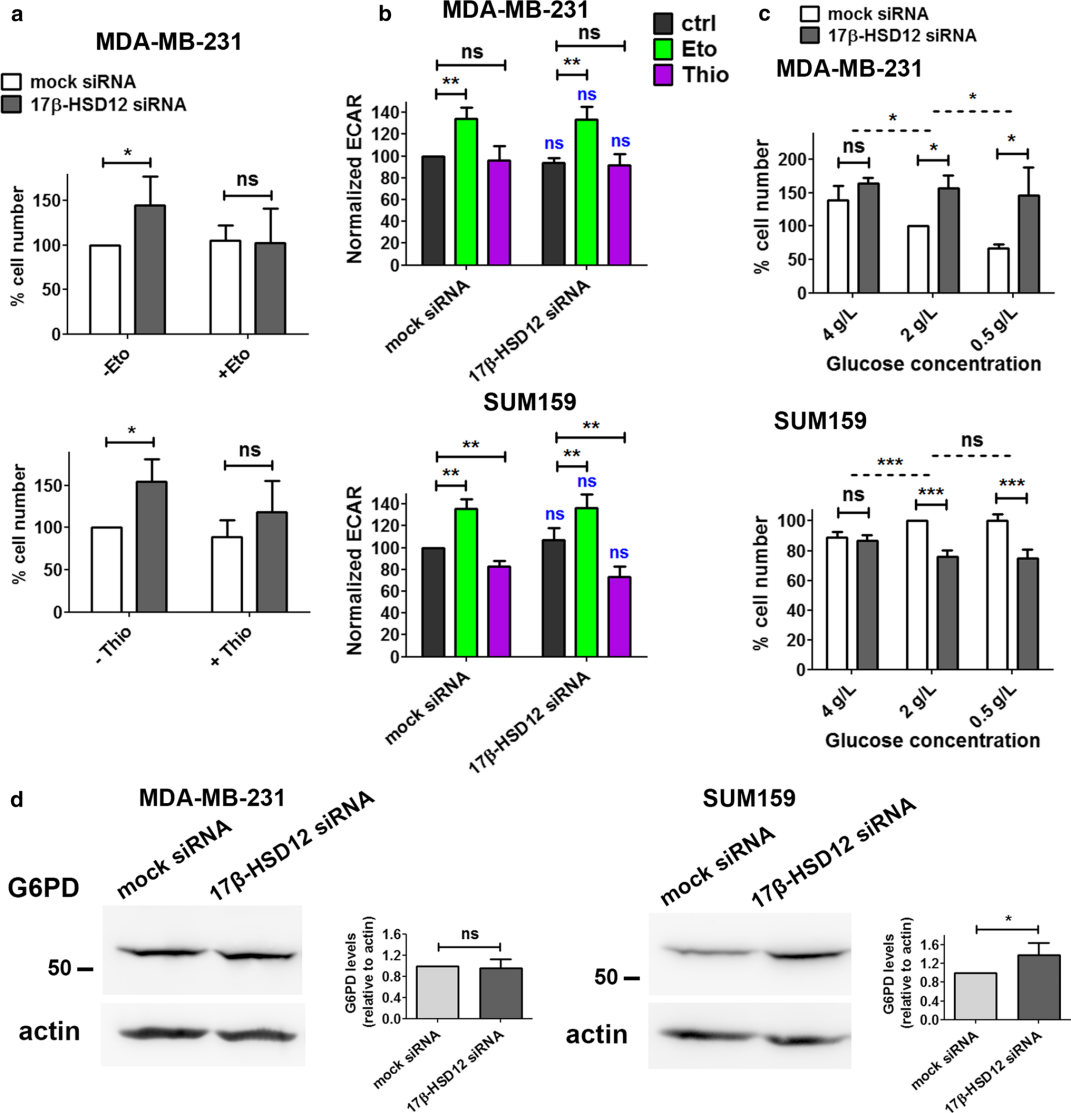
Effect of β-oxidation and glucose metabolism on the phenotype of 17β-HSD12 silencing. **a** Number of MDA-MB-231 cells counted 48 h post mock- or 17β-HSD12-siRNA transfection and 24 h after treatment with the inhibitor of mitochondrial β-oxidation Eto (top, *n* = 6) or peroxisomal β-oxidation Thio (bottom, *n* = 5) (mean ± SD, *p < 0.05, *ns* not significant). **b** ECAR was measured for MDA-MB-231 and SUM159 cells at baseline using the Seahorse XF96 analyzer during the experiments described in Fig. 5 (mean ± SD, *n* = 5, **p < 0.01, *ns* not significant). The statistical analysis depicted in blue characters compared the differences in the values between the corresponding mock siRNA and 17β-HSD12 siRNA samples. **c** Cells counted after knockdown of 17β-HSD12 in MDA-MB-231 (*n* = 4) and SUM159 (*n* = 5) cells and culturing in RPMI 1640 medium-containing 4 g/l, 2 g/l or 0.5 g/l glucose. The dotted lines represent the statistical comparison between the different mock siRNA/17β-HSD12 siRNA groups, whereas the solid lines constitute comparison between the indicated mock siRNA and 17β-HSD12 siRNA samples (mean ± SD, *p < 0.05, **p < 0.01, ***p < 0.01, *ns* not significant). **d** The levels of G6PD in MDA-MB-231 and SUM159 cells were assessed by western blot and quantified by densitometry (mean ± SD, *n* = 4, *p < 0.05, *ns* not significant)

Since Eto and Thio treatment reduced ATP production as well as spare respiratory capacity in MDA-MB-231 cells, we reasoned that β-oxidation could be vital in sustaining proliferation after 17β-HSD12 silencing in this cell line. Following downregulation of 17β-HSD12 expression and treatment of MDA-MB-231 cells with or without 1 μΜ Eto or Thio for 24 h, the 17β-HSD12 downregulation-dependent increased cell proliferation was suppressed after inhibition of mitochondrial or peroxisomal β-oxidation (Fig. 6a; Suppl. Figure 7b).

To further explore possible sources of energy and/or building blocks permitting increased proliferation and migratory potential of MDA-MB-231 cells after 17β-HSD12 silencing, we investigated whether glutamine metabolism, a major pathway in supporting cancer cell growth, was involved [33]. Our results showed that glutamine depletion for 48 h did not alter the 17β-HSD12 knockdown-dependent increase in proliferation and migration (Suppl. Figure 8a).

Another fundamental cellular energy source is glucose. Through glycolysis, glucose can be converted to pyruvate and then lactate, resulting in extrusion of protons into the extracellular medium. Pyruvate produced through glycolysis can also enter the TCA cycle in mitochondria, which produces NADH and FADH2 that are utilized in OXPHOS to generate more ATP. Alternatively, glucose can enter the pentose phosphate pathway (PPP) that produces NADPH as well as important building blocks, such as ribonucleotides. The first rate-limiting enzyme in this metabolic pathway is glucose 6-phosphate dehydrogenase (G6PD), which converts glucose 6-phosphate to 6-phosphogluconate, thereby generating NADPH. To gain more insight into the role of glucose after 17β-HSD12 knockdown, we first measured in real-time ECAR as a measure of glycolysis. Our results showed no significant difference in the levels of ECAR after 17β-HSD12 silencing in MDA-MB-231 or SUM159 cells (Fig. 6b). Inhibition of β-oxidation with Eto led to a compensatory increase in glycolysis in both cell lines, whereas Thio had no effect. However, Thio caused significant decrease in glycolysis after 17β-HSD12 knockdown only in SUM159 cells. To conclude whether glucose could play another role in growth of these cell lines following 17β-HSD12 silencing, we cultured SUM159 and MDA-MB-231 cells in RPMI 1640 medium-containing normal (2 g/l), higher (4 g/l), or lower (0.5 g/l) glucose concentration (Fig. 6c). Surprisingly, increasing the amount of glucose in MDA-MB-231 cells dampened the 17β-HSD12-knockdown phenotype, and decreasing glucose concentration led to a further accentuation of the phenotype. This was opposite to what we observed in SUM159 cells, where increased glucose rescued the reduction in proliferation following 17β-HSD12 silencing (Fig. 6c). No change in the 17β-HSD12-knockdown phenotype was observed at lower glucose concentrations. Interestingly, G6PD expression was found to be significantly increased in SUM159 after knockdown of 17β-HSD12, while it was unaltered in MDA-MB-231 cells (Fig. 6d). No difference in the expression of the second NADPH-producing enzyme of the PPP, phosphogluconate dehydrogenase (PGD) was observed in neither cell line after 17β-HSD12 knockdown (Suppl. Figure 8b). Taken together, the above results point towards a significant difference in glucose utilization between the two cell lines.

### Alteration in ER stress response and folding pathways after 17β-HSD12 knockdown

Long-chain fatty acids elongation takes place at the ER, the compartment where the cellular machineries for protein folding and stress response pathways reside. Nutrient depletion or defects in biosynthetic pathways induce a multitude of stress signals, although this has not been investigated for LCFAs. To address this, we first assessed the effects of 17β-HSD12 silencing on the UPR pathway. The UPR is activated through diverse stressors, including overload of protein folding demand in the ER, oxidative stress, and shortage in energy sources. It is well recognized that cancer cells exploit the UPR to sustain their high proliferation rates, resulting in constitutive overactivation of several UPR components [34]. The UPR consists of three main branches [35]; the protein kinase R-like ER kinase/eukaryotic initiation factor 2α (PERK/eIF2α), the inositol-requiring enzyme 1α/split X-box-binding protein 1 (IRE1α-Xbp1), and the activating transcription factor 6 (ATF6).

**Fig. 7:**
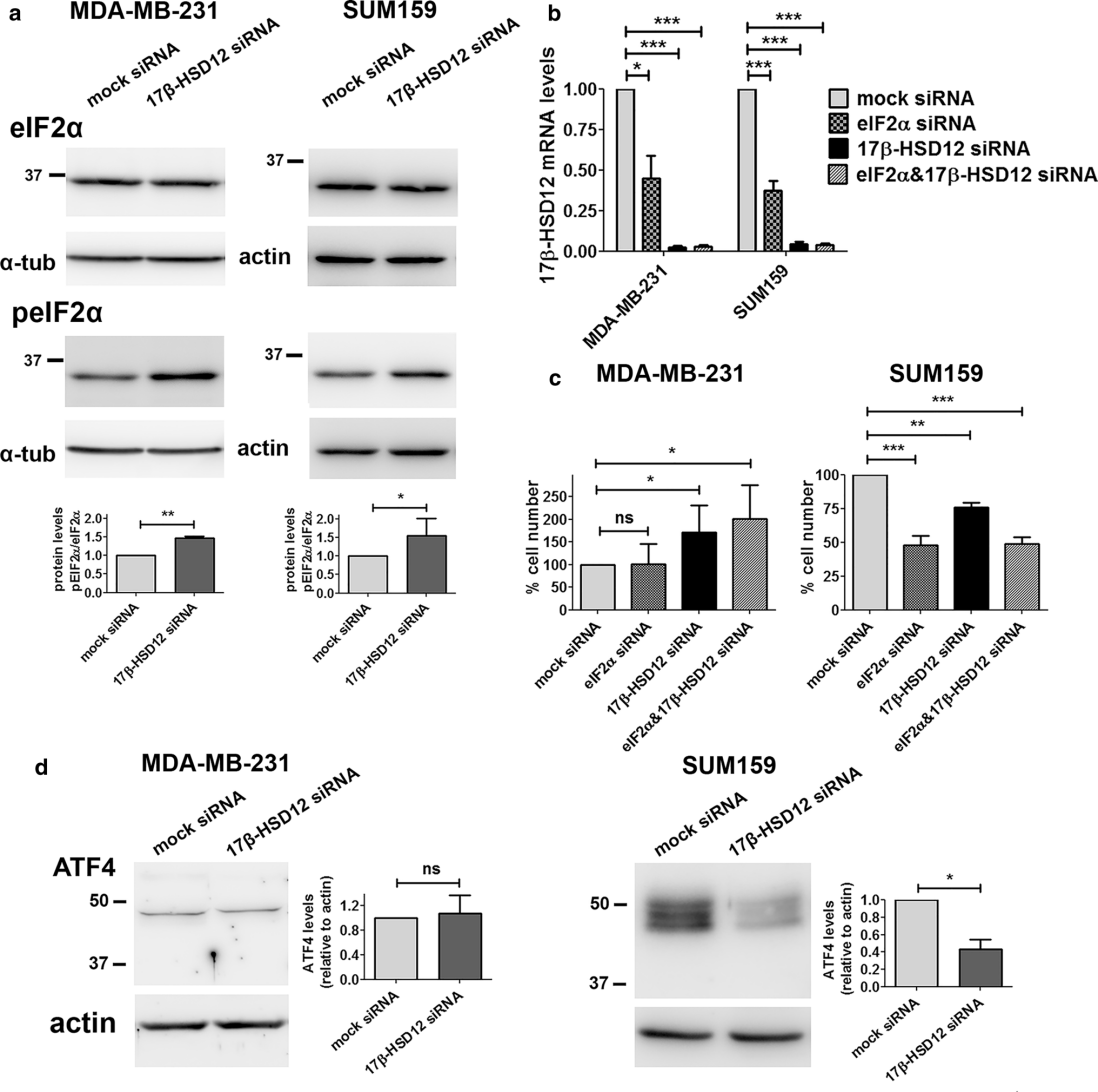
Alterations in the eIF2α/ATF4 pathway upon 17β-HSD12 downregulation. **a** Western blot analysis for protein expression of eIF2α and peIF2a after 17β-HSD12 knockdown in MDA-MB-231 (*n* = 6) and SUM159 (*n* = 3) cells. A representative blot and densitometry analysis of all independent experiments are shown. From the individual densities of eIF2α and peIF2, the rate of eIF2α phosphorylation was calculated (mean ± SD, *p < 0.05, **p < 0.01). **b** MDA-MB-231 and SUM159 cells were transfected with mock-, eIF2α-, 17β-HSD12- or eIF2α and 17β-HSD12-siRNA and the levels of 17β-HSD12 mRNA were measured by qPCR (mean ± SD, *n* = 4, *p < 0.05, ***p < 0.001). **c** MDA-MB-231 (*n* = 7) and SUM159 (*n* = 4) cells were treated as in Fig. 7b and cell number was evaluated in the different samples (mean ± SD, *p < 0.05, **p < 0.01, ***p < 0.001, *ns* not significant). **d** The expression of ATF4 protein was evaluated by western blot in MDA-MB-231 and SUM159 cells 48 h after 17β-HSD12 downregulation (mean ± SD, *n* = 4, *p < 0.05, *ns* not significant)

First, we downregulated 17β-HSD12 and performed western blot analysis for several UPR components at 48 h after knockdown in SUM159 and MDA-MB-231 cells. The first UPR branch is initiated by phosphorylation of PERK or other kinases, which in turn phosphorylate/activate eIF2α that selectively increases translation of specific mRNAs, including that of activating transcription factor 4 (ATF4). Activated ATF4 promotes transcription of target genes, and most commonly that of CCAAT/enhancer-binding protein homologous protein (CHOP, also known as DNA damage-inducible transcript 3 protein DDIT3). Following 17β-HSD12 downregulation in both SUM159 and MDA-MB-231 cells, we found increased phosphorylation of eIF2α (peIF2α), whereas total eIF2α protein levels remained stable (Fig. 7a). The total levels of PERK after 17β-HSD12 downregulation were unaffected (Suppl. Figure 9a); however, due to the lack of a specific antibody against pPERK, we could not conclude whether the increased phosphorylation of eIF2α was triggered by PERK or another kinase. The fact that eIF2α was activated irrespective of the differential effect on proliferation of 17β-HSD12 silencing in the two cell lines implies that this is a common cellular response upon defective LCFA biosynthesis. Notably, eIF2α downregulation led to a dramatic reduction of 17β-HSD12 mRNA levels (Fig. 7b). Knockdown of eIF2α in SUM159 cells caused a significant reduction in cell number and number of proliferating cells, mimicking 17β-HSD12 silencing (Fig. 7c; Suppl. Figure 9b). No effect on proliferation was observed after eIF2α knockdown in MDA-MB-231 cells, which could mean that the reduction in 17β-HSD12 levels after eIF2α downregulation is not sufficient to recapitulate the 17β-HSD12-knockdown phenotype in this cell line.

**Fig. 8:**
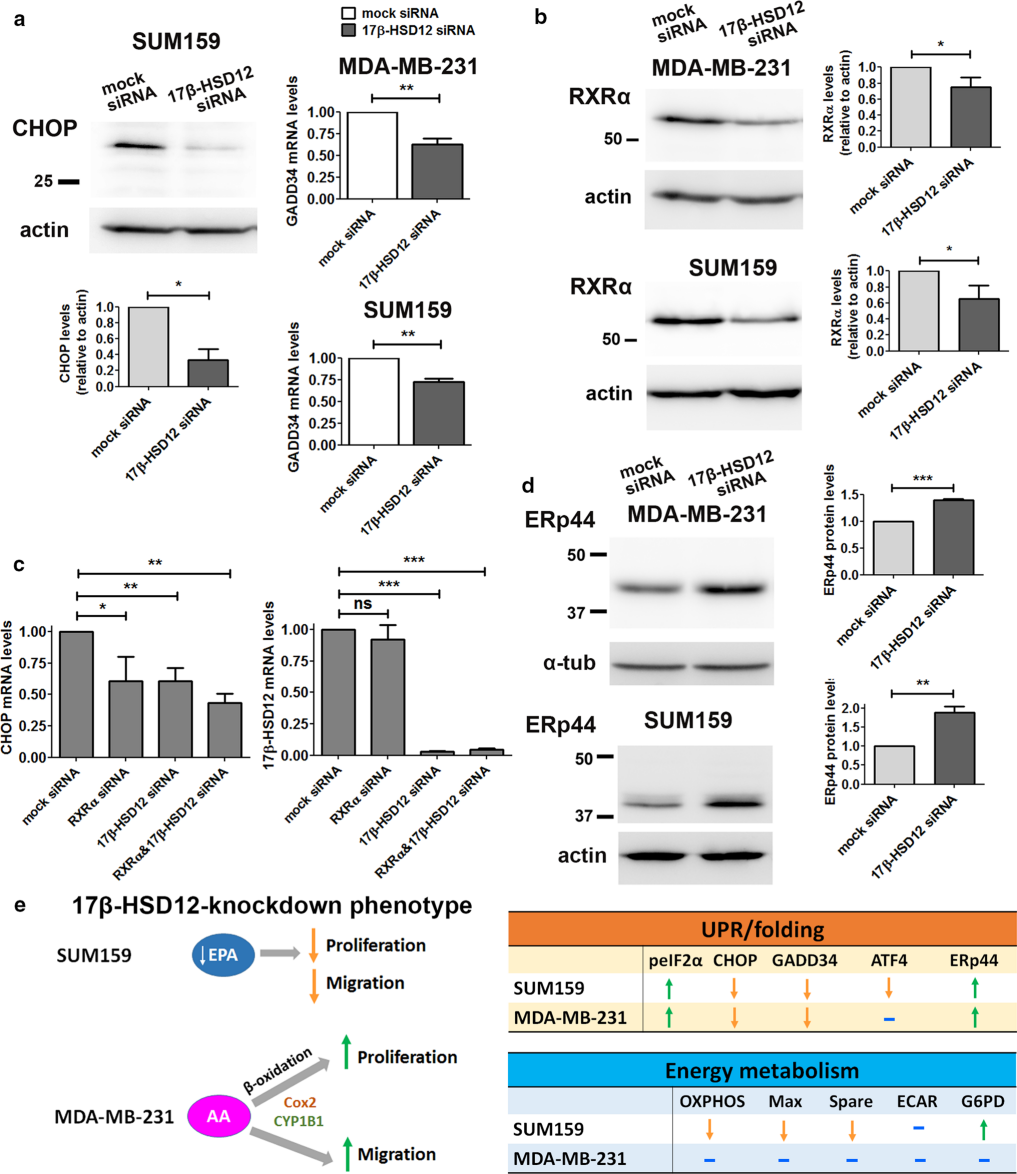
Effect of 17β-HSD12 knockdown on CHOP and ERp44 expression. **a** The protein levels of CHOP were assessed by western blot 48 h after 17β-HSD12 silencing in SUM159 cells. mRNA expression of the CHOP target GADD34 in MDA-MB-231 and SUM159 cells 48 h following 17β-HSD12 downregulation (mean ± SD, *n* = 4, *p < 0.05, **p < 0.01). **b** Expression of RXRα protein 48 h after 17β-HSD12 knockdown in MDA-MB-231 and SUM159 cells (mean ± SD, *n* = 4, *p < 0.05). **c** SUM159 cells were transfected with mock-, RXRα-, 17β-HSD12- or RXRα- and 17β-HSD12-siRNA, and CHOP or 17β-HSD12 mRNA expression was assessed by qPCR (mean ± SD, *n* = 5, *p < 0.05, **p < 0.01, ***p < 0.001, *ns* not significant). **d** The levels of the folding protein ERp44 were analyzed by western blot following 17β-HSD12 knockdown in MDA-MB-231 and SUM159 cells (mean ± SD, *n* = 5, **p < 0.01, ***p < 0.001). **e** Summary of the most prominent cellular changes observed in MDA-MB-231 and SUM159 cells after 17β-HSD12 knockdown. (Left panel) In SUM159 cells, 17β-HSD12 downregulation leads to decreased proliferation and migration, which can be restored by EPA supplementation. Aberration in synthesis of other FAs could additionally lead to the observed phenotype. In MDA-MB-231 cells, knockdown of 17β-HSD12 leads to increased AA metabolism through the COX and CYPB1 pathways that promote proliferation and migration. The augmented proliferation after 17β-HSD12 knockdown is supported by energy and/or building blocks provided by β-oxidation. (Right panel) Comparison of the alterations in UPR/folding and energy metabolism in SUM159 and MDA-MB-231 cells following 17β-HSD12 silencing

Although ATF4 is a target of eIF2a, 17β-HSD12 siRNA transfection led to decreased ATF4 protein levels in SUM159 cells, while it had no effect in MDA-MB-231 cells (Fig. 7d). This demonstrates that although eIF2α is activated upon 17β-HSD12 silencing, it does not stabilize ATF4 protein as expected, which further suggests that it constitutes a counterbalance mechanism. In addition, in SUM159 cells ATF4 ran in western blot as a triplet, likely representing different degrees of phosphorylation, whereas only one band could be detected in the MDA-MB-231 cells (Fig. 7d), indicating different degrees of ATF4 activation between the two cell lines. Furthermore, the mRNA and protein levels of the ATF4 target CHOP significantly declined in both cell lines following 17β-HSD12 silencing (Fig. 8a; Suppl. Figure 9c and d). The levels of the downstream CHOP target GADD34 (growth arrest and DNA damage-inducible protein 34), the phosphatase that dephosphorylates eIF2α, were reduced, which can explain the increased eIF2α phosphorylation (Fig. 8a). Since no difference in ATF4 following 17β-HSD12 downregulation was found in MDA-MB-231 cells, CHOP expression is possibly regulated by transcription factors other than ATF4, at least in this cell line. CHOP has been shown to be a retinoid-responsive gene [36]. Therefore, we investigated whether its expression is regulated by retinoid X receptor alpha (RXRα), since this receptor has been linked with cell proliferation control, and we found it to be abundantly expressed in both SUM159 and MDA-MB-231 cells. To examine whether the reduced CHOP levels following 17-HSD12 downregulation could be mediated by reduced expression of RXRα, we determined RXRα levels. In accordance with our hypothesis, RXRα levels were reduced at the mRNA and protein level in both cell lines after 17β-HSD12 silencing (Fig. 8b; Suppl. Figure 9d). Moreover, downregulation of RXRα led to decreased CHOP mRNA levels, comparable to that observed after 17β-HSD12 silencing, while it had no impact on 17β-HSD12 mRNA expression (Fig. 8c). Regarding the other UPR pathways, we found no difference in the levels of ATF6 or sXBP1 (the active form of XBP1 that initiates IRE1α-mediated signals) in neither cell line 48 h following 17β-HSD12 knockdown (Suppl. Figure 9e and f). Consequently, these UPR branches were not pursued further.

Since the UPR pathway is closely related to protein folding, we also examined a potential influence of 17β-HSD12 downregulation on several protein folding components. Among the proteins involved in folding are the heat-shock responsive proteins, including Grp78 and Grp94, which were not affected by silencing of 17β-HSD12 (Suppl. Figure 10a). One of the most important families of folding-control proteins is the protein disulfide isomerases (PDI). Although the expression of two members of this family, namely PDI and ERp72, was not changed following 17β-HSD12 downregulation (Suppl. Figure 10b), there was a significant increase in ERp44 expression at the mRNA and protein levels in both SUM159 and MDA-MB-231 cells (Fig. 8d; Suppl. Figure 10c). Given that eIF2α was overactivated after 17β-HSD12 knockdown, we examined whether it could be involved in stabilizing the ERp44 mRNA. In contrast to this hypothesis, knockdown of eIF2α led to a small rise in the levels of ERp44 (Suppl. Figure 10d).

## Discussion

The rapid growth of cancer cells requires a high amount of energy and macromolecules, including carbohydrates, proteins, lipids, and nucleic acids. These demands are largely met by metabolic rewiring, which entails de novo synthesis of cellular building blocks. The role of FAs in malignant transformation is highly relevant, with a multitude of alterations in FA synthesis and break down through β-oxidation reported in several types of cancer [37]. Besides, certain FAs act as signaling molecules and are involved in metabolic adaptation. This work addressed the role of LCFA biosynthesis for cancer cells. Of the four enzymatic entities participating in LCFA synthesis, 17β-HSD12 has garnered attention regarding its role in cancer through studies yielding conflicting results. While some studies reported an association of 17β-HSD12 with more aggressive tumor progression and poor outcome [21–23], others found a suppressive role [25]. Using different breast cancer cells, we showed that silencing of 17β-HSD12 either increases or decreases cancer cell proliferation and migration depending on the cellular context. Importantly, two triple-negative breast cancer cell lines, MDA-MB-231 and SUM159, exhibit opposite phenotypes with increased and decreased proliferation and migration, respectively, after 17β-HSD12 knockdown, emphasizing the heterogeneity of breast cancer cells (results summarized in Fig. 8e). In a previous report, 17β-HSD12 downregulation caused a dramatic decrease in proliferation after culturing the cells in serum-free medium during knockdown, a condition that severely hinders cell growth [21]. Our experiments were performed under normal culture conditions, highlighting that even under sufficient energy supply, impaired LCFA synthesis affects cancer cell proliferation and invasiveness. The effect of 17β-HSD12 knockdown on cell migration was followed by changes in the levels of MMPs that were not accompanied by altered cell adhesion. This suggests that they likely represent offset mechanisms to initial alterations in the molecular network controlling cell substrate attachment after 17β-HSD12 silencing.

To understand the differential impact of 17β-HSD12 downregulation, we compared MDA-MB-231 and SUM159 cells. Differences in the culture medium could not explain the observed divergent phenotypes of the two cell lines following 17β-HSD12 silencing; therefore, we explored the expression of enzymes participating in LCFA synthesis, focusing on ω-6 and ω-3 PUFAs. Since HACDs 1–4 show tissue but not substrate specificities, and only one TER and one KAR enzyme have been identified so far [38, 39], we tested whether distinct expression of ELOVLs with different substrate specificities directs differential FA synthesis in the two cell lines. Expression of ELOVL2, which is indispensable for the elongation of PUFAs with 22 carbon atoms [40, 41] (Fig. 1b), was not detected in the two cell lines, suggesting that this process does not take place in these cells and resembling the situation in proliferating T cells [42]. In addition, we found that ELOVL5 mRNA and protein expression was lower in SUM159 compared to MDA-MB-231 cells. ELOVL5 elongates 18 carbon PUFAs as well as PUFAs with 20 carbon atoms such as AA and EPA, and several studies indicated a preference of this enzyme for ω-3 FAs [40, 42–45]. This could mean that at the low ELOVL5 expression in SUM159 cells, the activity is directed towards EPA over AA elongation. The FADS enzymes also display substrate specificities in PUFA elongation (Fig. 1b). Although we found much lower FADS2 mRNA levels in SUM159 than in MDA-MB-231 cells, western blot revealed similar protein expression in both cell lines. This discrepancy between mRNA and protein levels could be due to a higher rate of FADS2 mRNA translation in SUM159 compared with MDA-MB-231 cells, resulting in comparable protein levels. Nevertheless, because protein expression is not significantly different between the two cells, FADS2 unlikely is responsible for the observed differences in their proliferation after 17β-HSD12 knockdown. Future experiments should include a complete proteomics profiling in the two cancer cell lines to unveil key differences in other enzymes participating in synthesis of saturated and monounsaturated LCFAs.

According to one of our hypotheses, the production of different LCFAs in MDA-MB-231 and SUM159 cells could account for the different phenotypes after 17β-HSD12 knockdown. Indeed, supplementation with EPA following 17β-HSD12 silencing rescued the decreased proliferation and migration of SUM159 cells. It is unclear whether this is a direct effect or caused through formation of downstream metabolites. AA or DHA supplementation failed to revert the 17β-HSD12-knockdown phenotype, supporting an EPA-specific effect. This finding was unexpected, since EPA was associated with suppression of breast cancer cell growth [46]. However, EPA treatment was shown to increase proliferation of RAW264.7 macrophages and of a B-lymphocyte cell line, which in the latter was attributed to the absence of EPA-to-DHA conversion [47, 48]. Due to swift conversion of EPA to DHA in certain cell types, the effects of the two FAs cannot always be distinguished. In MDA-MB-231 cells, AA exacerbated the 17β-HSD12 siRNA-induced rise in cell proliferation, whereas EPA and DHA had no impact. We reasoned that this might be due to AA conversion towards eicosanoids promoting cancer cell growth and invasiveness. Indeed, blocking the activities of COX2 and CYP1B1 that are involved in this metabolism reverted the effect of 17β-HSD12 knockdown on MDA-MB-231 cells, indicating that in this cell line, elongation of AA competes with its processing through eicosanoid synthesis pathways. Of note, CYP1B1 is not expressed in SUM159 cells, and inhibiting COX2 did not alter the proliferation phenotype after 17β-HSD12 silencing. Importantly, 17β-HSD12 silencing led to a change in AA levels exclusively in MDA-MB-231 cells. Downstream metabolism of AA through COX and CYP enzymes may, consequently, promote cancer cell growth and migratory potential. Taken together, our results suggest that the metabolism of AA and EPA is implicated in the observed phenotypes after 17β-HSD12 silencing in MDA-MB-231 and SUM159 cells, respectively. In addition, differential AA metabolism emerges as a crucial divergent point between the two cell lines, which offers an explanation for the differential 17β-HSD12-knockdown phenotype. After AA release from membrane phospholipids by phospholipase A2, cell proliferation was found to be induced via activation of the serine–threonine kinase Akt [49–51]. Surprisingly, our results showed reduced phosphorylation of Akt at Ser473 at 48 h after 17β-HSD12 downregulation. This finding may be explained by a negative feedback loop after a transient increase in Akt phosphorylation or alternative survival pathways in this cell system.

Since our analysis covered only a small fraction of the possible FAs affected by 17β-HSD12 downregulation, future lipidomics analysis should provide a comprehensive picture of the changes following defects in LCFA synthesis in different cancer cell lines. Reliable methods for detailed and time-effective analysis of lipidomes in cells and various organisms have lately been developed [52]. Our preliminary experiments in SUM159 cells indicate a shift from longer to shorter chain LCFAs upon 17β-HSD12 knockdown (data not shown).

Since FAs are hardly soluble in aqueous solutions, for our experiments, FAs were conjugated with BSA, as previously described [53, 54]. Before entering cells, FAs dissociate from BSA and are intracellularly transported in a free form either through diffusion or protein-mediated transport [55]. In addition, free FAs can bind to G protein-coupled receptors at the cell surface, thereby exerting signaling effects that can affect tumor growth and chemoresistance [56, 57]. The most important receptors mediating LCFA action are GPR40 and GPR120, whereby GPR40 was shown to potentiate some of the effects of free FAs in MDA-MB-231 cells [58, 59]. Thus, it would be interesting to explore whether the phenotypes observed after 17β-HSD12 knockdown and treatment of cells with FAs are influenced by these receptors.

By altering the metabolic phenotype of cancer cells, impaired LCFA synthesis could mediate effects on cell proliferation upon 17β-HSD12 silencing. Our results revealed that 17β-HSD12 knockdown decreased ATP production through OXPHOS in SUM159 cells, whereas MDA-MB-231 cells appeared resistant. It should be noted that these experiments were performed 48 h after 17β-HSD12 siRNA transfection, a time point when SUM159 cell proliferation was not yet significantly reduced, implying that the decreased OXPHOS precedes impairments of proliferation and suggesting a causative effect. Inhibition of peroxisomal β-oxidation with Thio markedly decreased maximal respiration and spare respiratory capacity, which may have crucial consequences when cells grow under conditions requiring mobilization of their energy reserves. Strikingly, blockage of peroxisomal β-oxidation led to an increased proton leak. Proton leak is considered a protective mechanism against reactive oxygen species (ROS) generation and, in turn, ROS are known to promote proton leak [60]. The observed rise in proton leak could be a consequence of increased ROS after Thio treatment or a reaction to increased ROS formation. Blocking peroxisomal β-oxidation could augment the amount of free LCFAs, which have been shown to carry protons across the mitochondrial membrane, by shuffling between the protonated and non-protonated states [61, 62]. Inhibiting mitochondrial β-oxidation severely blocks OXPHOS in both cell lines, indicating that breakdown of FAs is a major contributor to mitochondrial ATP production. This is mainly mediated by acetyl-CoA production, the ultimate product of β-oxidation, which can readily enter the TCA cycle that subsequently fuels the electron transport chain [63]. Since we found β-oxidation to be a prevailing factor in sustaining ATP production through OXPHOS, we examined whether it mediates the increased proliferation observed after 17β-HSD12 downregulation in MDA-MB-231 cells. In line with our hypothesis, inhibition of mitochondrial or peroxisomal β-oxidation attenuated the 17β-HSD12-knockdown effect on the proliferation of these cells. Although peroxisomal β-oxidation does not directly yield ATP, further breakdown of FAs in mitochondria links the two β-oxidation pathways.

A key metabolite supporting cancer cell growth is glucose. Based on live measurements of ECAR, we concluded that 17β-HSD12 downregulation does not affect glycolysis in neither cell line. However, the expression of G6PD, the enzyme catalyzing the first and rate-limiting enzyme in the PPP of glucose metabolism, was upregulated upon silencing of 17β-HSD12 only in SUM159 cells. Through the enzymatic conversion of glucose 6-phosphate, G6PD generates NADPH, an essential co-factor for the synthesis of short-chain FAs by FAS in the cytosol. The importance of the PPP for tumor cells has recently started to be recognized [64]. Silencing of G6PD MCF7 breast cancer cells reduced lipid synthesis and proliferation [65]. Importantly, in the present study, increased glucose concentration in the culture medium fully restored proliferation after 17β-HSD12 knockdown in SUM159 cells. In contrast, increased glucose concentration suppressed the increased proliferation upon 17β-HSD12 downregulation in MDA-MB-231 cells, while decreased glucose concentration further enhanced proliferation. These results imply that the cellular pathways in which glucose participates are competitive to the ones that MDA-MB-231 cells utilize to boost proliferation after 17β-HSD12 knockdown. This may be explained by competition between glycolysis and the β-oxidation pathway that sustains high proliferation after 17β-HSD12 silencing. Although both glycolysis and β-oxidation produce acetyl-CoA that fuels the TCA cycle, β-oxidation is more efficient by providing a higher number of carbon atoms per molecule [66]. β-oxidation of FAs can thus generate more ATP molecules than glycolysis. In accordance, we demonstrated that the majority of OXPHOS in both cancer cell lines is fueled by β-oxidation.

It has recently been recognized that the UPR pathway is of paramount importance for tumor cell growth, due to its role in maintaining excessive bioenergetic needs and growth under hypoxic and nutrient-limited conditions. Importantly, the UPR is constantly alert to changes in lipid metabolism. For example, membrane lipid composition directly triggers the UPR, irrespective of proteostasis, and perturbations in ER membrane lipid composition activate several UPR components, including PERK and IRE1 [67, 68]. Interestingly, we found that 17β-HSD12 downregulation induced the phosphorylation/activation of eIF2α. Downregulation of eIF2α drastically decreased 17β-HSD12 levels, implying that 17β-HSD12 expression is controlled by eIF2α, and mimicked some of the effects observed after 17β-HSD12 knockdown, such as the decreased proliferation of SUM159 cells. The increased phosphorylation of eIF2α after 17β-HSD12 knockdown could thus represent a compensatory mechanism. Upon activation, eIF2α induces a global reduction in protein synthesis, but specifically enhances translation of certain mRNAs through the control of translation from upstream open reading frames (sORF) [69]. The only mRNA that has been unequivocally shown to be subjected to such regulation is that of ATF4. Since ATF4 was reduced when eIF2α was activated in the SUM159 cells, and we did not observe any general attenuation of protein synthesis in neither cell line after 17β-HSD12 knockdown, we conclude that activation of eIF2a is a response to the decreased 17β-HSD12 mRNA levels. In this context, it would be interesting to investigate whether 17β-HSD12 is an mRNA stabilized by eIF2α and whether other lipid metabolizing enzymes could be subject to this type of regulation.

Another UPR component modified by 17β-HSD12 knockdown in SUM159 and MDA-MB-231 cells was CHOP, exhibiting a decrease at both mRNA and protein levels. CHOP is a transcription factor that can be activated by both the eIF2a/ATF4 and ATF6 branches of the UPR, and has been associated with ER stress-induced apoptosis [70]. However, CHOP is abundantly expressed in MDA-MB-231 and SUM159 cells at steady state, pointing towards an alternative function of this factor in these cells. In addition, CHOP expression was reduced after 17β-HSD12 knockdown in both cell lines, independently of the proliferation rate. Recent evidence suggests that CHOP possesses a dual function; it regulates apoptosis after extensive stressful stimuli, such as amino acid deprivation, but also induces the expression of autophagy-related genes at initial stages of starvation [71, 72]. Therefore, CHOP expression in tumor cells at basal levels may control autophagy, a cellular process with a crucial role for survival of many tumors [73]. Among others, autophagy encompasses lipid degradation to free FAs (lipophagy) that fuels β-oxidation [74]. It is thus possible that CHOP sustains growth of tumor cells through maintaining signals for optimal autophagy levels, including lipid recycling. In addition, studies in CHOP^−/−^ mice have shown that it directly regulates the expression of master regulators of lipid metabolism, such as sterol regulatory element-binding proteins (SREBPs) and the peroxisome proliferator-activated receptor alpha (PPARα) [75]. This favors a role of CHOP in sustaining cancer cell growth through the control of metabolic genes. The mechanism of how CHOP expression was reduced after 17β-HSD12 downregulation remains to be discovered. In liver, FXR/RXR heterodimers regulate CHOP expression; therefore, we tested the potential involvement of RXRα, which is abundantly expressed in MDA-MB-231 and SUM159 cells. 17β-HSD12 knockdown diminished RXRα mRNA and protein levels, and RXRα downregulation led to decreased CHOP levels, as also observed after 17β-HSD12 downregulation. Although a direct link cannot be drawn from these experiments, our results suggest RXRα as a candidate regulator of CHOP expression.

Examination of folding-control proteins revealed activation of ERp44 as a common denominator of 17β-HSD12 silencing in MDA-MB-231 and SUM159 cells. This increase of ERp44 did not seem to be controlled by eIF2α; silencing eIF2α led to a small increase in ERp44 expression, which could be due to the decreased 17β-HSD12 levels upon eIF2α silencing. These data further support that eIF2α is upstream of 17β-HSD12 and controls its expression. ERp44 is an interesting member of the PDI family, since it shunts between the ER, ER–Golgi intermediate compartment (ERGIC), and cis-Golgi [76, 77]. It is dormant in the ER but active in more distal compartments where it binds to its client proteins, releasing them only when their maturation is complete. Among the proteins falling into ERp44 quality control are multimeric proteins, such as immunoglobin M (IgM) and adiponectin [78–80]. Increased ERp44 expression may be a consequence of high demand in folding of specific proteins or triggered by other stimuli, such as redox status imbalance provoked by 17β-HSD12 silencing. ERp44 knockdown induced apoptosis in HeLa cells, while ERp44 expression inhibited migration in the human non-small lung cancer cell line A549 through a pathway involving inositol 1,4,5-trisphosphate receptor type 2 (IP3R2) [81, 82]. Given that the ERp44 increase after 17β-HSD12 downregulation was followed by decreased and increased proliferation/migration in SUM159 and MDA-MB-231 cells, respectively, it is likely that ERp44 upregulation is an early event following impaired LCFA synthesis. Its specific involvement in tumor cell homeostasis warrants further investigation.

High 17β-HSD12 expression has been associated with poor prognosis in breast and ovarian cancer patients [21–23]. At the same time, 17β-HSD12 was found to be a prognostic marker gene for favorable and adverse outcome in patients with kidney and liver cancer, respectively [25]. Our study explains these apparently contradicting results, since we showed that the phenotype after 17β-HSD12 silencing could diverge, depending on the cellular context. The complex biosynthetic pathway of LCFA synthesis, the pleiotropic effects of the produced FAs, as well as the differential responses of different types of cells to impaired biochemical processes, could all underlie the divergent phenotype upon downregulation of 17β-HSD12 expression. The notion that genes can be categorized as cancer promoting or suppressing is slowly shifting, after the realization that genes can either promote or suppress the growth of cells depending on the cancer type, underscoring the heterogeneous nature of tumor cells. To our knowledge, this is the first study reporting the cellular changes following impaired LCFA synthesis due to 17β-HSD12 silencing and sets a basis for further understanding of this aspect of lipid metabolism for malignant transformation of cells.

### Electronic supplementary material

Below is the link to the electronic supplementary material.

Supplementary material 1 (PDF 1939 kb)
