# Supplementary material for: Impact of 17β-HSD12, the 3-ketoacyl-CoA reductase of long-chain fatty acid synthesis, on breast cancer cell proliferation and migration
Source: Cell Mol Life Sci. 2019 Jul 13;77(6):1153–75. doi: 10.1007/s00018-019-03227-w (PMC7109200; doi:10.1007/s00018-019-03227-w)
Supplement: Supplementary file 1 — Supplementary material 1 (PDF 1939 kb) [file 18_2019_3227_MOESM1_ESM.pdf]

Electronic supplementary material

Impact of 17β-HSD12, the 3-ketoacyl-CoA reductase of long-chain fatty acid synthesis, on breast cancer cell proliferation and migration

Maria Tsachaki[1], Pirmin Strauss[1], Anja Dunkel[1], Hana Navrátilová[1,a], Natasa Mladenovic[1] and Alex Odermatt[1*]

[1] Division of Molecular and Systems Toxicology, Department of Pharmaceutical Sciences, University of Basel, Klingelbergstrasse 50, 4056 Basel, Switzerland

[a] Current address: Department of Biochemical Sciences, Faculty of Pharmacy in Hradec Králové, Charles University, Heyrovskeho 1203, 500 05 Hradec Kralove, Czech Republic.

Supplementary Figure 1

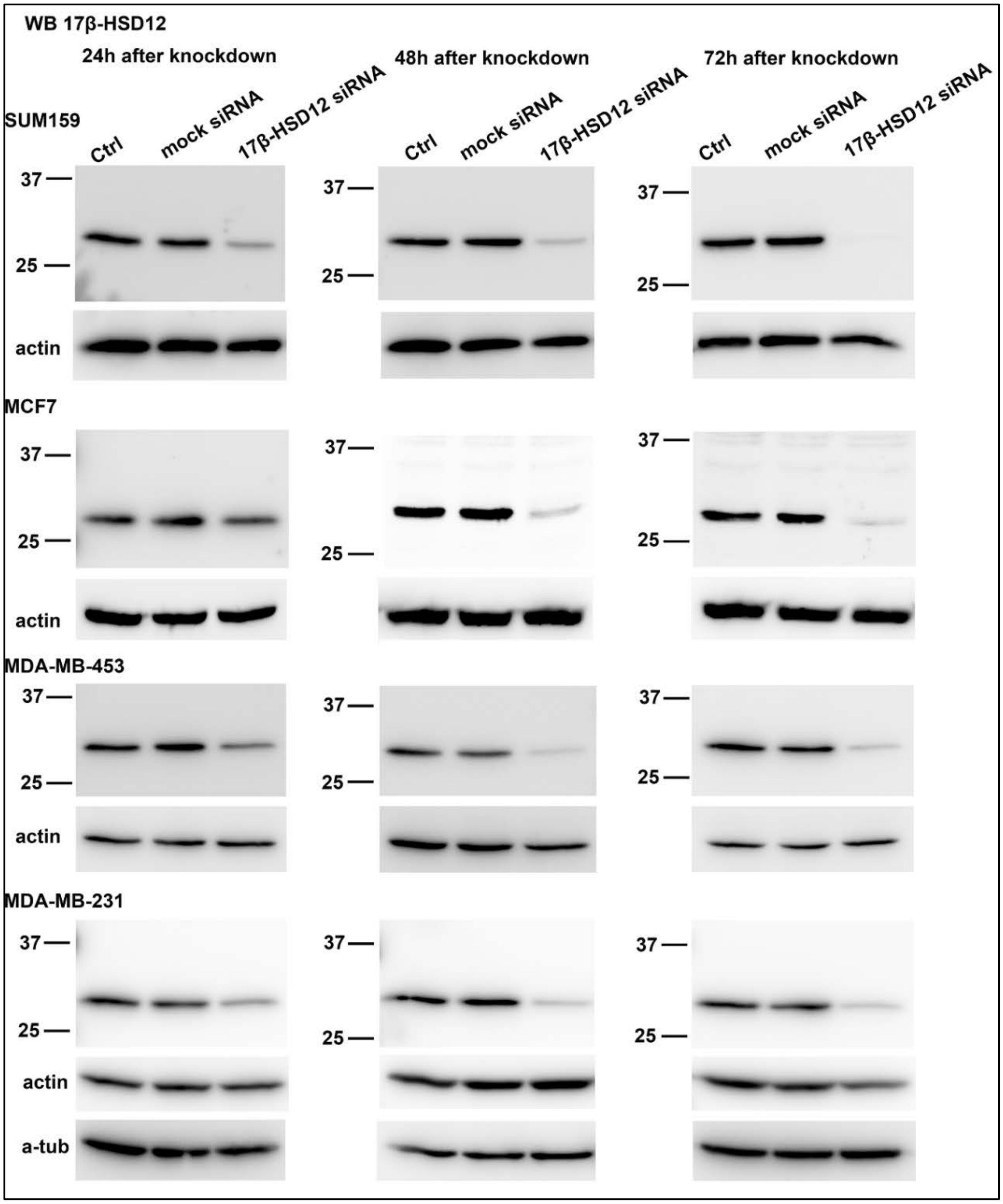

Supplementary Figure 1. Efficiency of 17β-HSD12 downregulation in SUM159, MCF7, MDA-MB-453 and MDA-MB-231 cells. 17β-HSD12 protein expression was analyzed by western blot 24 h, 48 h and 72 h after transfection with mock or 17β-HSD12 siRNAs. To evaluate potential changes in expression due to transfection, untreated cells (ctrl) were also analyzed. Actin or PPIA served as loading control. In MDA-MB-231 cells, on account of consistent small reduction in actin expression 72 h after 17β-HSD12 downregulation compared to mock and ctrl samples, α-tubulin was additionally used as loading control.

Supplementary Figure 2

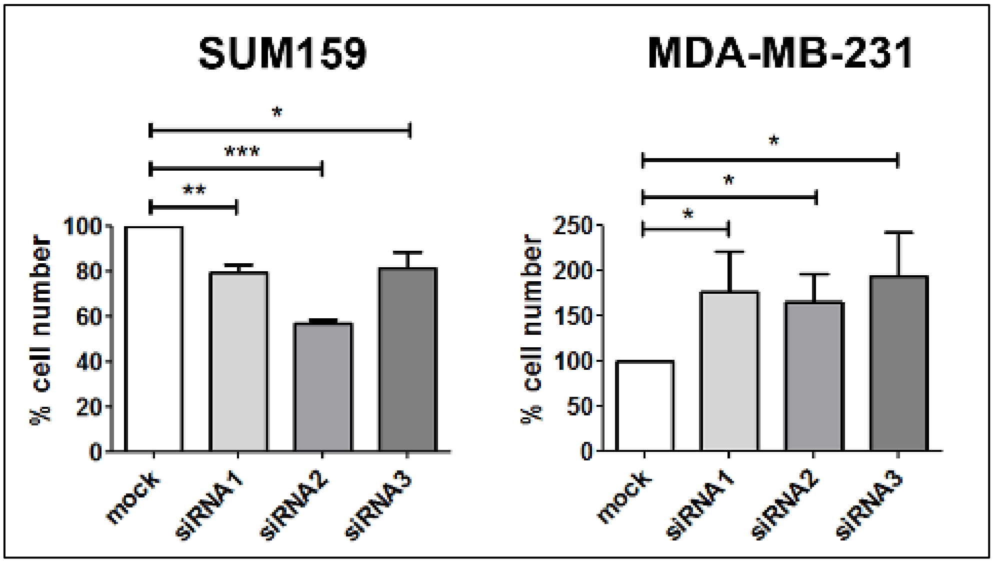

Supplementary Figure 2. Effect of 17β-HSD12 knockdown with different siRNAs on proliferation of SUM159 and MDA-MB-231 cells. The expression of 17β-HSD12 was downregulated using three different siRNAs (siRNA1, siRNA2, siRNA3) in SUM159 and MDA-MB-231 cells. Cell number was measured with Hoechst staining and high-content imaging at 72 h or 48 h post-transfection for SUM159 and MDA-MB-231 cells, respectively. All values were statistically compared to mock-transfected cells (mean ± SD, SUM159 n=3, MDA-MB-231 n=4, *p<0,05, **p<0,01, ***p<0,001).

Supplementary Figure 3

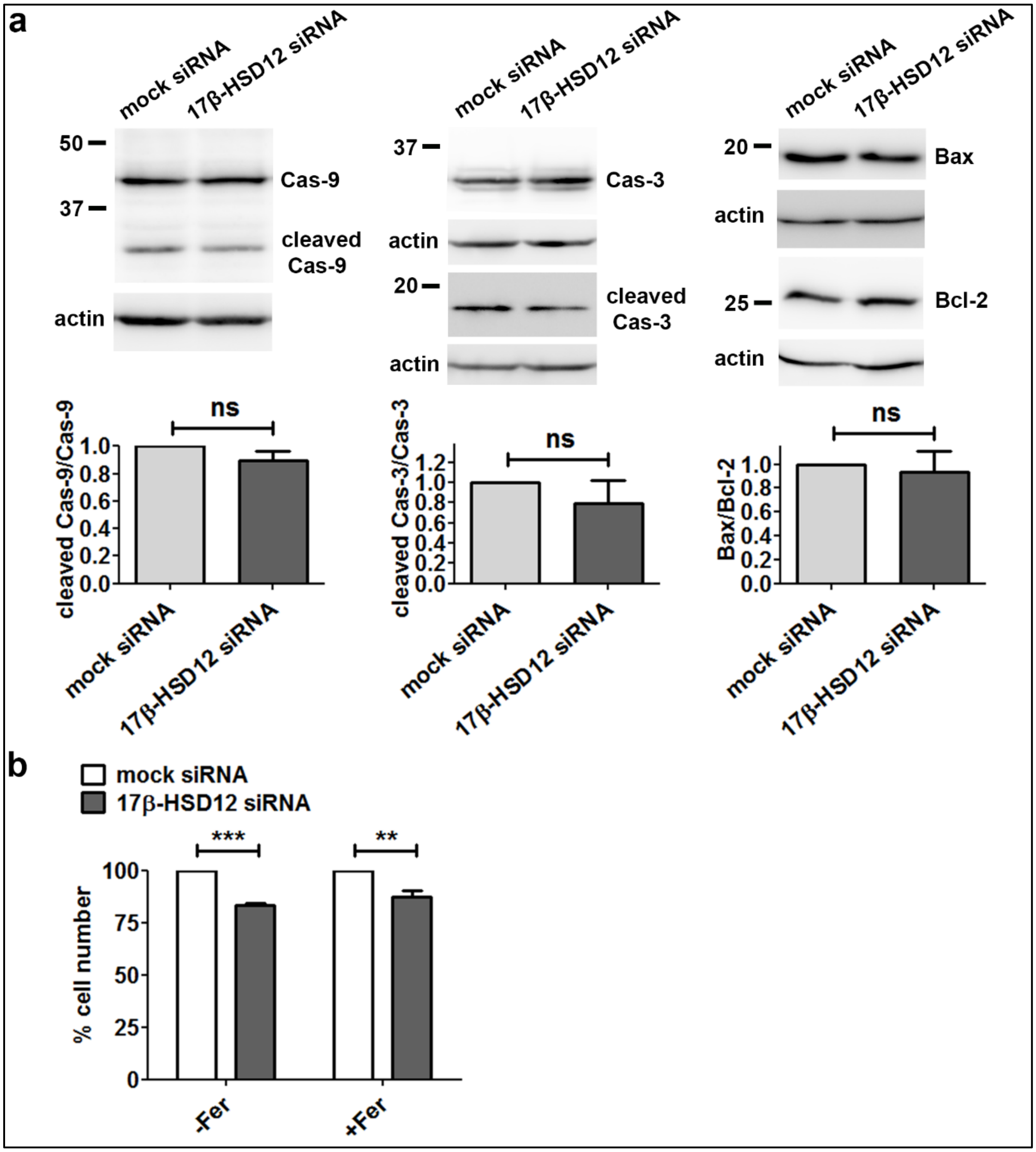

Supplementary Figure 3. Evaluation of apoptosis and ferroptosis upon 17β-HSD12 silencing in SUM159 cells.

a Expression of Cas-9, cleaved Cas-9, Cas-3, cleaved Cas-3, Bax and Bcl-2 at 48 h following 17β-HSD12 downregulation. The ratios of cleaved Cas-9/Cas-9, cleaved Cas-3/Cas-3 and Bax/Bcl-2 were statistically compared between mock- and 17β-HSD12-siRNA-transfected cells (mean ± SD, cleaved Cas-9/Cas-9 n=4, cleaved Cas-3/Cas-3 n=3, Bax/Bcl-2 n=4, ns=not significant).

b SUM159 cells were transfected with mock- or 17β-HSD12- siRNAs and treated with 1 μM of the ferroptosis inhibitor ferrostatin (Fer) 24 h later. Cell number was measured 72 h after siRNA delivery (mean ± SD, n=3, **p<0,01, ***p<0,001).

Supplementary Figure 4

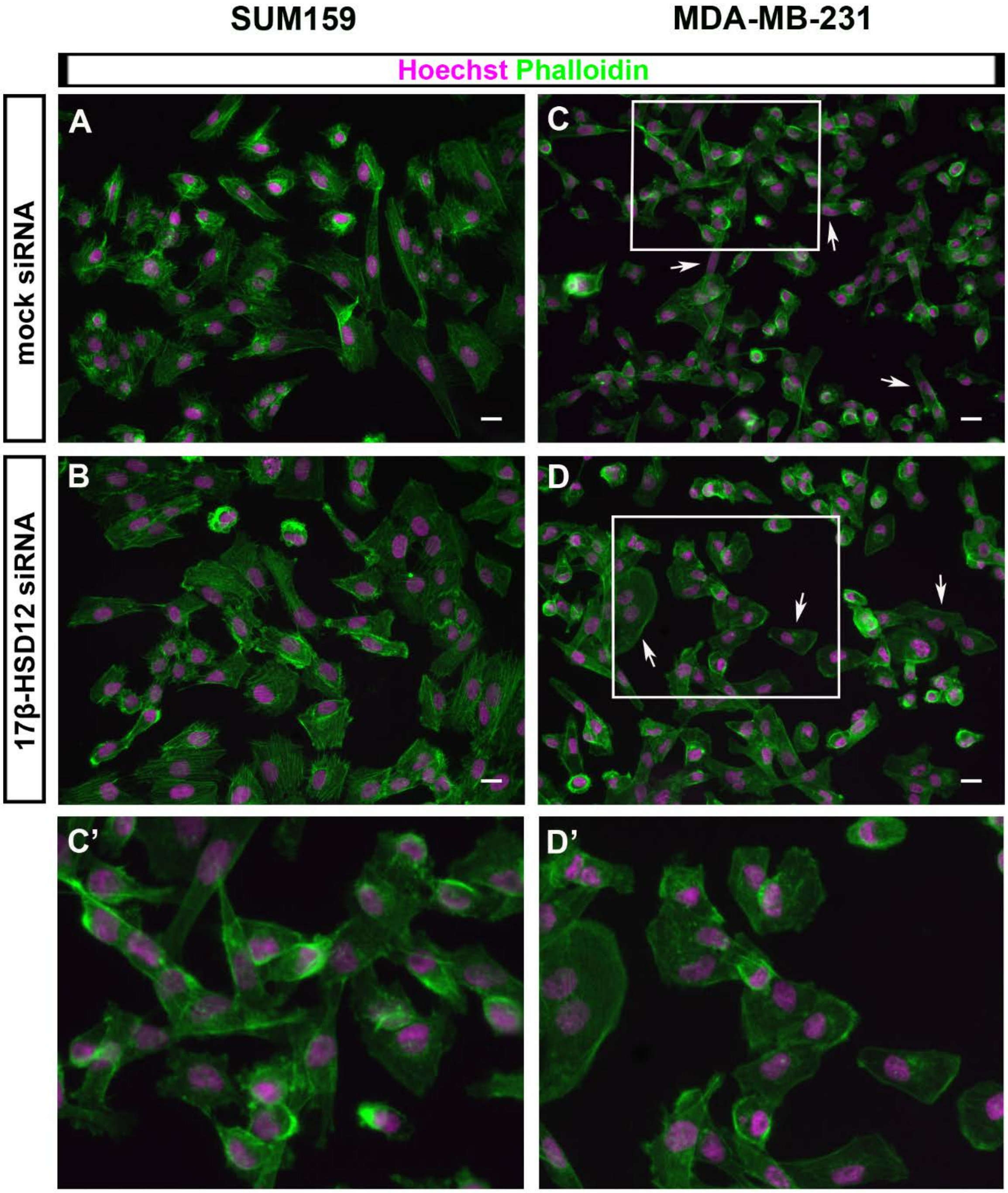

Supplementary Figure 4. Cellular pattern of actin filaments upon 17β-HSD12 silencing. SUM159 and MDA-MB-231 cells were stained with phalloidin-FITC (green) and Hoechst-33342 (magenta) 48 h after mock- or 17β-HSD12- siRNA transfection. Cells where the difference in actin pattern between mock- or 17β-HSD12- siRNA-transfected MDA-MB-231 cells is evident are pointed with arrows. C' and D' are higher magnifications of the inlets in panels C and D, respectively. Three independent experiments were performed and representative images are shown. Scale bars correspond to 20 μm.

Supplementary Figure 5

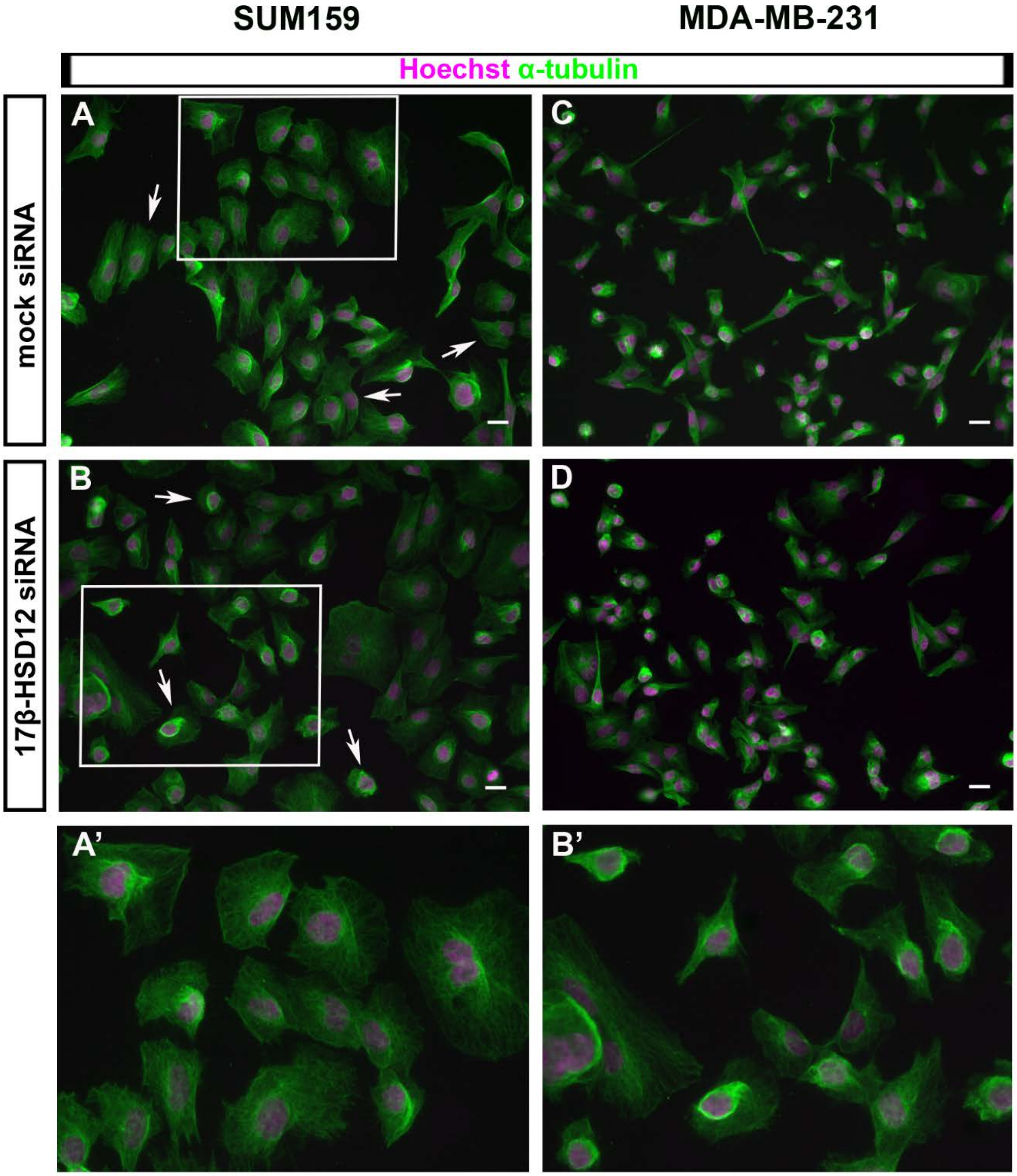

Supplementary Figure 5. Structure of microtubules after 17β-HSD12 knockdown. Immunofluorescence staining of SUM159 and MDA-MB-231 cells, 48 h after transfection with mock- or 17β-HSD12-siRNAs, with an antibody against α-tubulin (green). Nuclei were stained with Hoechst-33342 (magenta). In SUM159 cells, arrows mark cells that are representative of the difference in tubulin pattern between mock- or 17β-HSD12-siRNA-transfected cells. A’ and B’ are higher magnifications of the inlets in panels A and B, respectively. Representative images from 3 independent experiments are shown. Scale bars correspond to 20 µm.

Supplementary Figure 6

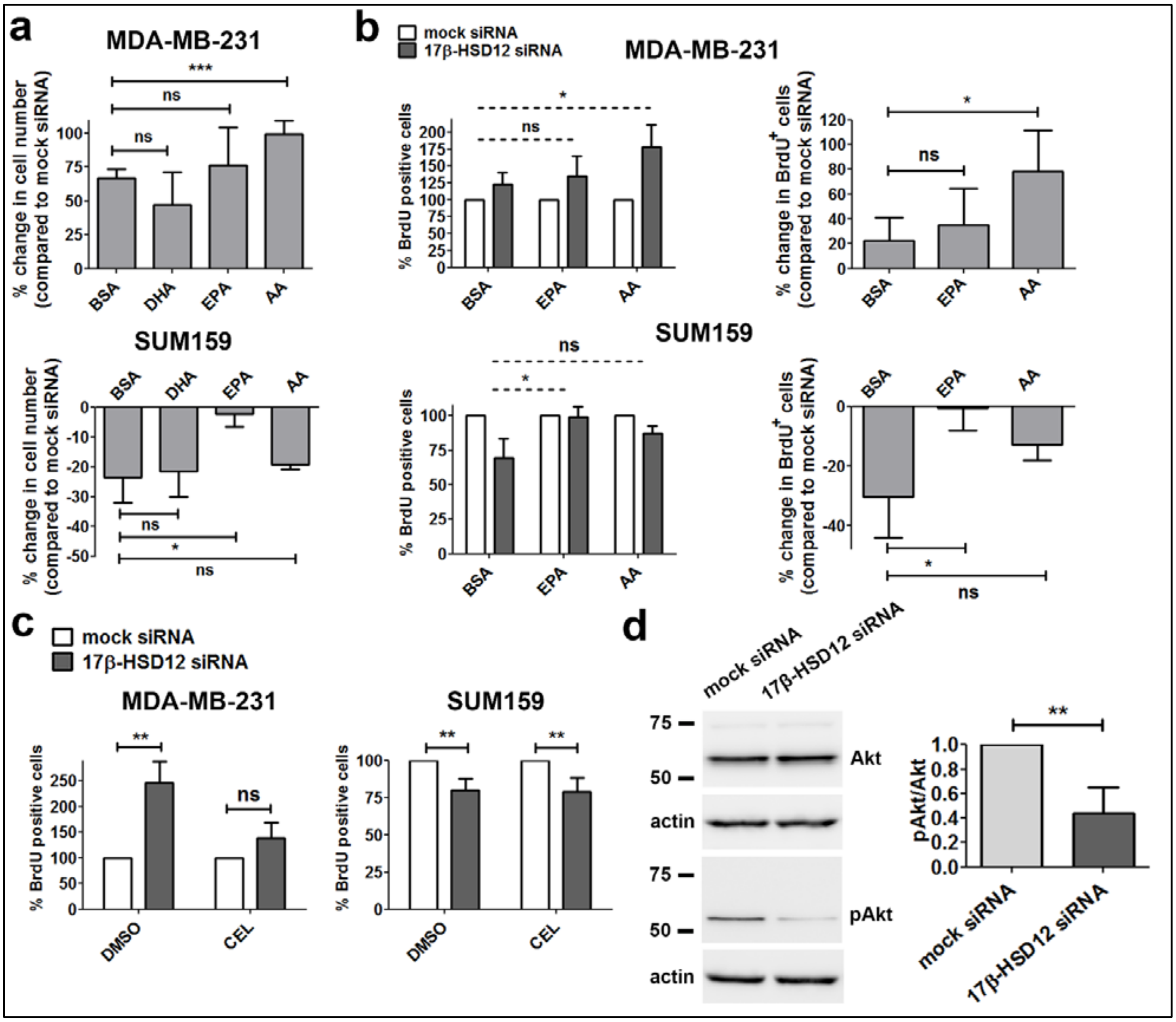

Supplementary Figure 6. Effect of different FAs and downstream molecular pathways in cell proliferation of MDA-MB-231 and SUM159 cells following 17β-HSD12 downregulation.

a Data of Figure 4a is presented as percent of change in cell number in the MDA-MB-231 and SUM159 cell lines.

b MDA-MB-231 and SUM159 cells were transfected with mock- or 17β-HSD12-siRNA and treated with BSA or BSA-conjugated EPA and AA. At 48 h after knockdown, cell proliferation was determined with BrdU staining and high-content imaging. The statistical analysis shown (left, dotted lines) compared the degree of difference between the various mock and 17β-HSD12 siRNA groups. The percent of change in BrdU-positive cells is also depicted and statistically compared (right, solid lines) for each cell line (mean ± SD, MDA-MB-231 n=5, SUM159 n=4, *p<0,05, ns: not significant).

c Percentage of proliferating MDA-MB-231 and SUM159 cells was assessed after BrdU incorporation 48 h after transfection with mock- or 17β-HSD12- siRNA and 24 h after treatment with DMSO or 1 μM Celecoxib (CEL) (mean ± SD, n=5, **p<0,01, ns: not significant).

d The levels of Akt and pAkt (Ser 473) protein were analyzed 48 h following 17β-HSD12 downregulation in MDA-MB-231 cells and the ratio of pAkt/Akt calculated and statistically compared to the mock siRNA control (mean ± SD, n=5, **p<0,01).

Supplementary Figure 7

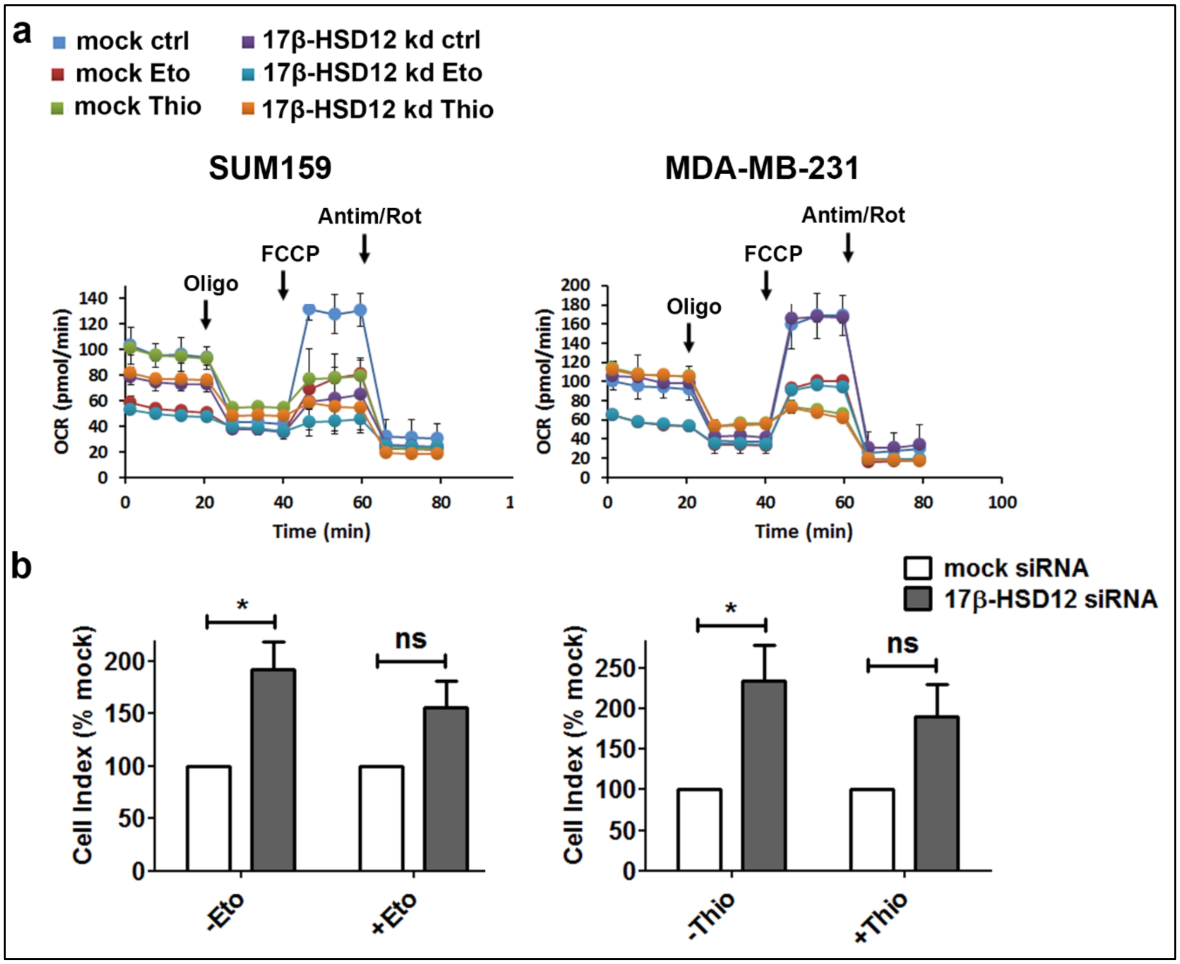

Supplementary Figure 7. Energy metabolism after 17β-HSD12 knockdown.

a Measurement of OCR over time in a representative experiment. The values represent the mean of six technical replicates and the error bars the standard deviation of the replicate values. Arrows indicate the time points of injection of the different compounds (oligomycin, FCCP, antimycin/rotenone).

b Cell index derived from RTCA measurements of cell proliferation at approximately 48 h after downregulation of 17β-HSD12 in MDA-MB-231 cells and treatment with the inhibitors of mitochondrial β-oxidation Etomoxir (Eto) or peroxisomal β-oxidation Thioridazine (Thio).

Supplementary Figure 8

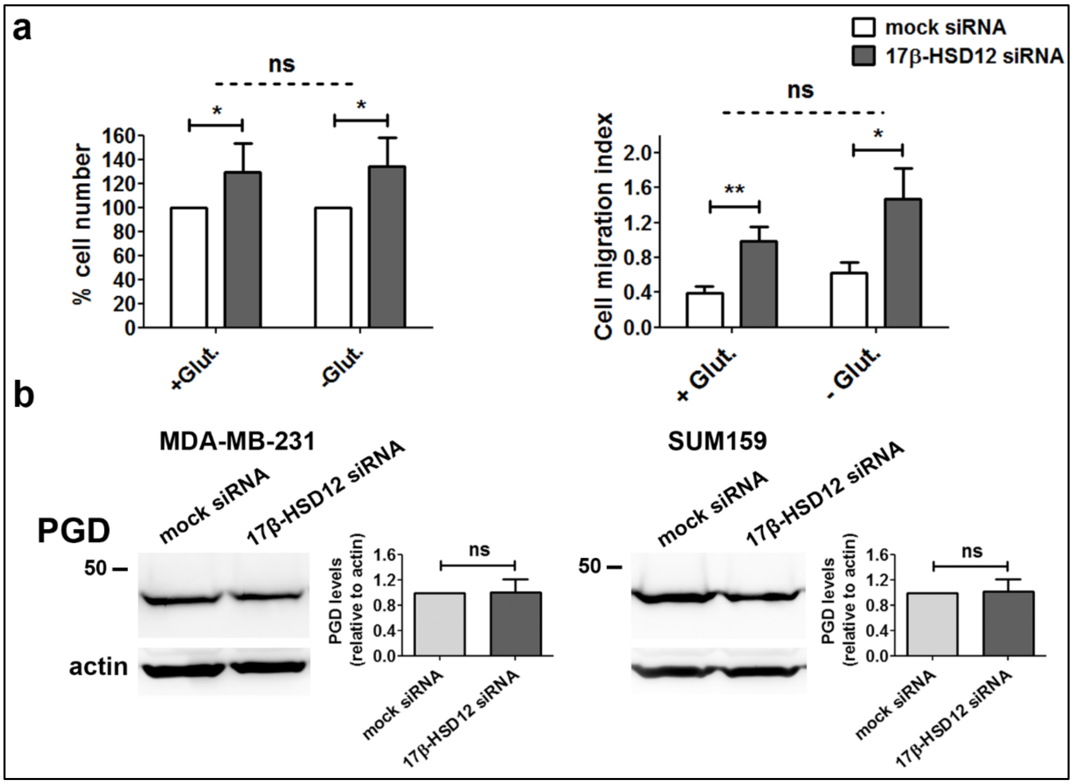

Supplementary Figure 8. Involvement of glutamine and glucose energy metabolism pathways in the phenotype observed after 17β-HSD12 silencing.

a MDA-MB-231 cells were transfected with mock- or 17β-HSD12-siRNA and cultured in RPMI 1640 containing or not glutamine (+Glut., -Glut.). At 48 h post-transfection, cell number was assessed (n=5), as well as the cells that migrated in a trans-well assay using the xCELLigence RTCA instrument (n=4). The dotted lines show whether the ability of 17β-HSD12 downregulation to increase proliferation or migration was significantly different between the +Glut and –Glut groups (mean ± SD, *p<0,05, **p<0,01, ns: not significant).

b Protein expression of phosphogluconate dehydrogenase (PGD) in MDA-MB-231 and SUM159 cells 48 h after 17β-HSD12 downregulation, along with densitometry analysis of the independent experiments (mean ± SD, n=4, ns: not significant).

Supplementary Figure 9

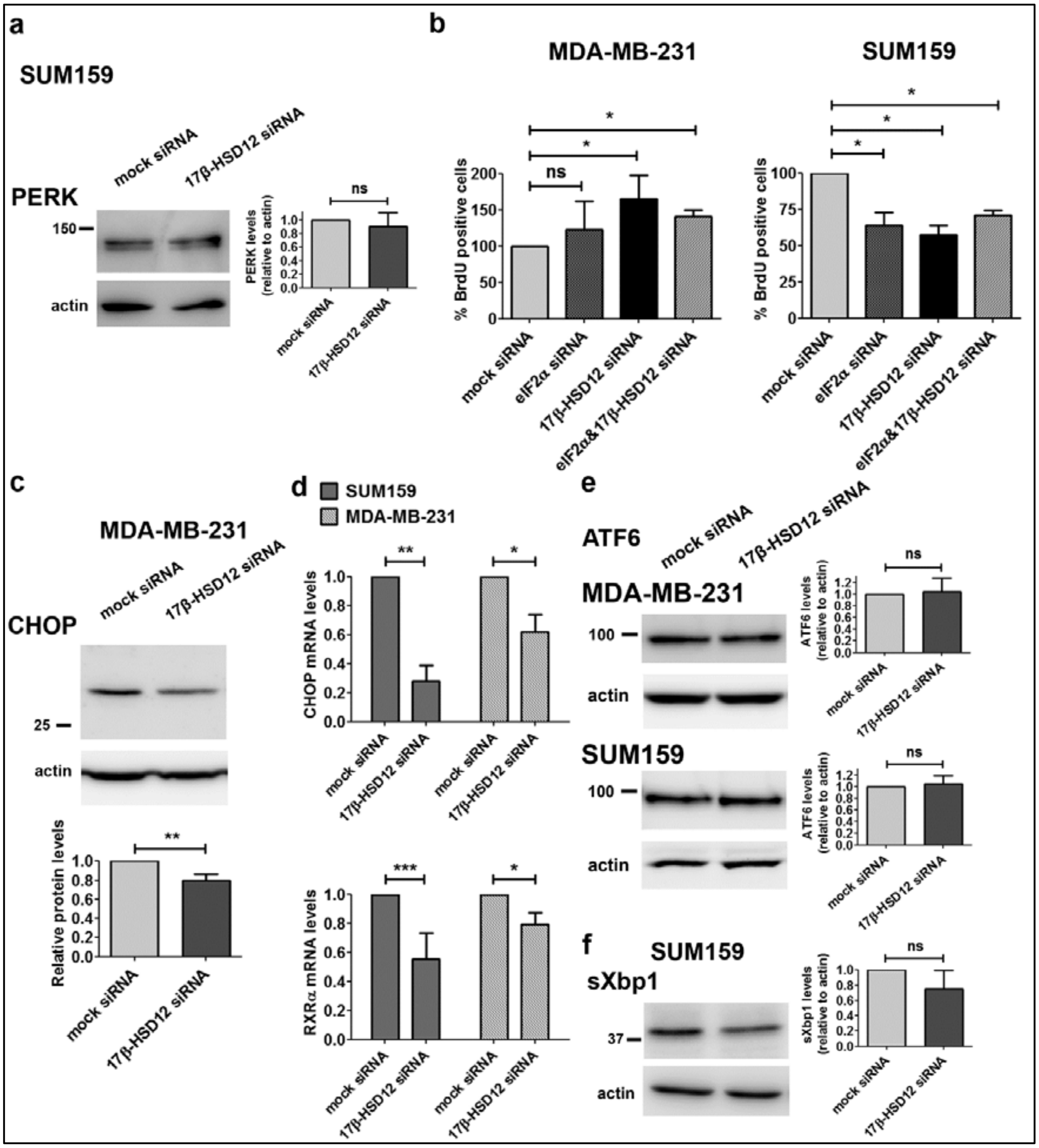

Supplementary Figure 9. Alterations in UPR components following 17β-HSD12 silencing.

a The protein levels of PERK were analyzed in SUM159 cells following 17β-HSD12 silencing (mean ± SD, n=4, ns: not significant).

b The experiment described in Fig. 7c was repeated in order to assess cell proliferation following BrdU incorporation at 48 h after 17β-HSD12 downregulation (mean ± SD, MDA-MB-231 n=5, SUM159 n=3, *p<0,05, ns: not significant).

c Similar to **Fig. 8a** for MDA-MB-231 cells.

d CHOP and RXRα mRNA levels measured with qPCR 48 h post mock- or 17β-HSD12-siRNA transfection in MDA-MB-231 and SUM159 cells (mean ± SD, n=4, *p<0,05, **p<0,01, ***p<0,001).

e ATF6 protein expression was assessed by western blot in MDA-MB-231 and SUM159 cells 48 h after knockdown of 17β-HSD12 (mean ± SD, n=4, ns: not significant).

f The expression of sXbp1 was analyzed by western blot 48h following 17β-HSD12 silencing in SUM159 cells (mean ± SD, n=3, ns: not significant).

Supplementary Figure 10

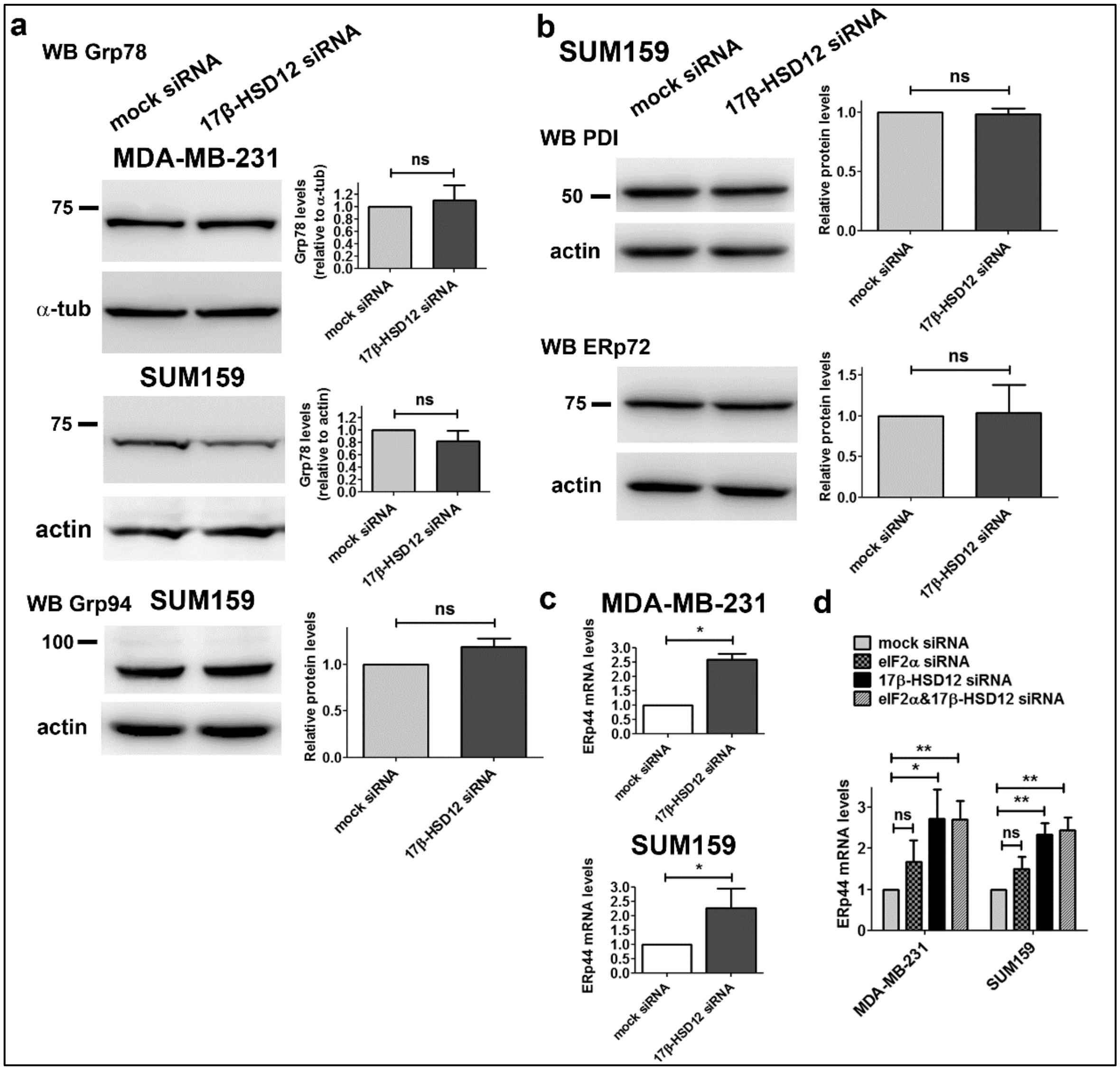

Supplementary Figure 10. Impact of 17β-HSD12 downregulation on proteins involved in folding control.

a (top) Grp78 protein levels were examined 48 h after 17β-HSD12 downregulation in MDA-MB-231 and SUM159 cells (mean ± SD, n=4, ns: not significant). **(bottom)** SUM159 cells were transfected with mock- or 17β-HSD12-siRNA and the expression of Grp94 was analyzed by western blot (mean ± SD, n=4, ns: not significant).

b Expression of the folding-control proteins PDI and ERp72 was analyzed by western blot in SUM159 cells following 17β-HSD12 knockdown. A representative image and the densitometry analysis of the independent experiments are shown (mean ± SD, n=4, ns: not significant).

c ERp44 mRNA levels 48 h after mock- or 17β-HSD12-siRNA transfection in MDA-MB-231 and SUM159 cells (mean ± SD, n=4, *p<0,05).

d Similar to **Fig. 7b** for ERp44 mRNA levels.
